# Multigram-Scale Synthesis of 2,5-Dideoxy-2,5-imino-d-mannitol (DMDP) and 2,5-Dideoxy-2,5-imino-d-glucitol (DGDP) from d-Fructose and l-Sorbose Using a Regioselective Appel Reaction

**DOI:** 10.1021/acs.joc.4c02667

**Published:** 2025-02-05

**Authors:** Peter Sunde-Brown, Gavin J. Miller, Todd A. Houston

**Affiliations:** † Institute for Glycomics, Griffith University, Gold Coast Campus, Southport 4215 , Queensland, Australia; ‡ School of Chemical and Physical Sciences and Centre for Glycoscience, Keele University, Keele, Staffordshire ST5 5BG, U.K.; § School of Environment and Science, Griffith University, Gold Coast Campus, 4215 Gold Coast, Queensland, Australia

## Abstract

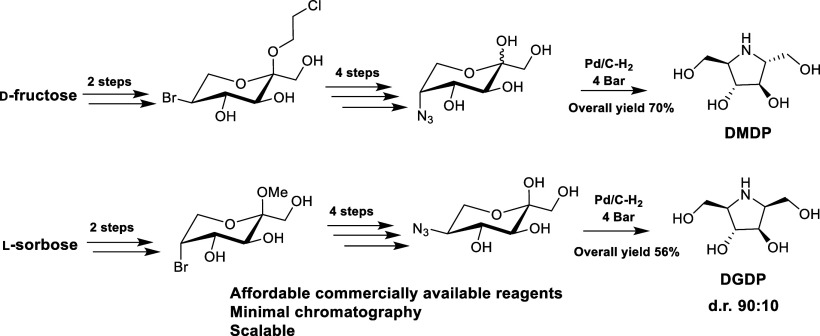

We report a practical 5 g scale stereoselective synthesis of the valuable iminosugar DMDP from d-fructose in only 7 synthetic steps and in a 70% overall yield, which doubles previously reported yields. This process requires only two chromatographic purification steps, taking advantage of a regioselective Appel reaction. The regioselective reaction has also been applied on a similar scale to prepare the C-2 diastereomer of DMDP, DGDP, from l-sorbose in 7 steps (two purifications) and 56% overall yield, albeit with diminished diastereomeric purity (d.r. 90:10). The C-5 regioselectivity has also been illustrated on d-psicose and d-tagatose, making this an attractive method for preparing pyrrolidine iminosugars or 5-thiosugars from ketopyranoses.

## Introduction

**Figure 1:**
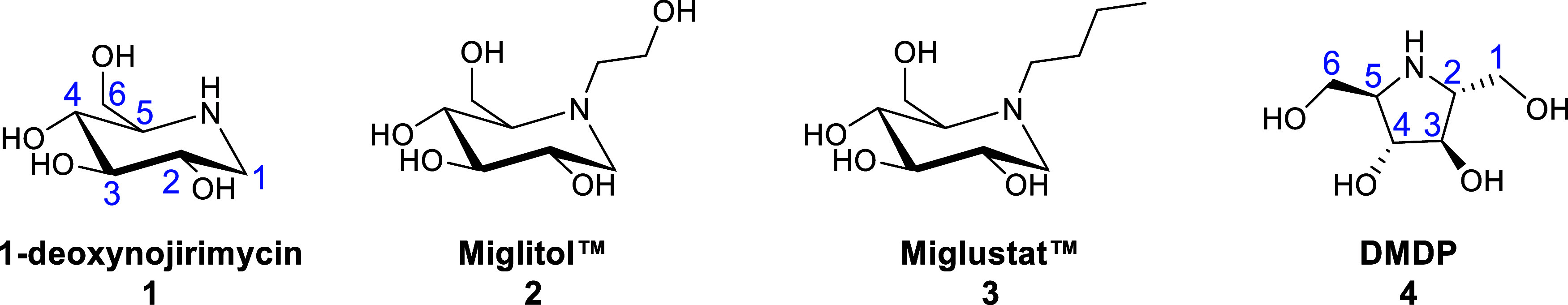
Naturally occurring iminosugars 1-DNJ (**1**) and DMDP (2,5-dideoxy-2,5-imino-d-mannitol) (**4**) and *N*-alkyl-derived drugs Miglitol (*N*-hydroxyethyl-1-deoxynojirimycin) (**2**) and Miglustat (*N*-butyl-1-deoxynojirimycin) (**3**).

Iminosugars are a valuable class of naturally occurring sugar mimetics which inhibit a variety of biologically important enzymes, such as glycosyl hydrolases, by mimicking the oxocarbenium ion transition state.^1^ Due to their potent biological activity, iminosugar-derived drugs have become a valuable class of therapeutics to treat a variety of diseases, most notably the two piperidine derivatives based on the 1-deoxynojirimycin (1-DNJ, **1**) scaffold, which treat type-2 diabetes mellitus^2^ (Miglitol, **2**) and type-1 Gaucher disease^2^ (Miglustat, **3**) by inhibiting glycoprocessing enzymes (Figure 1).

Development of pyrrolidine-derived iminosugars, such as 2,5-dideoxy-2,5-imino-d-mannitol (DMDP, **4**) and its derivatives, has been comparatively underexplored, presumably due to the increased difficulty in preparation compared to **1**, which can be prepared on a kilogram scale from 2,3,4,5-tetra-*O*-benzyl-d-glucopyranose.^3^

**Scheme 1:**
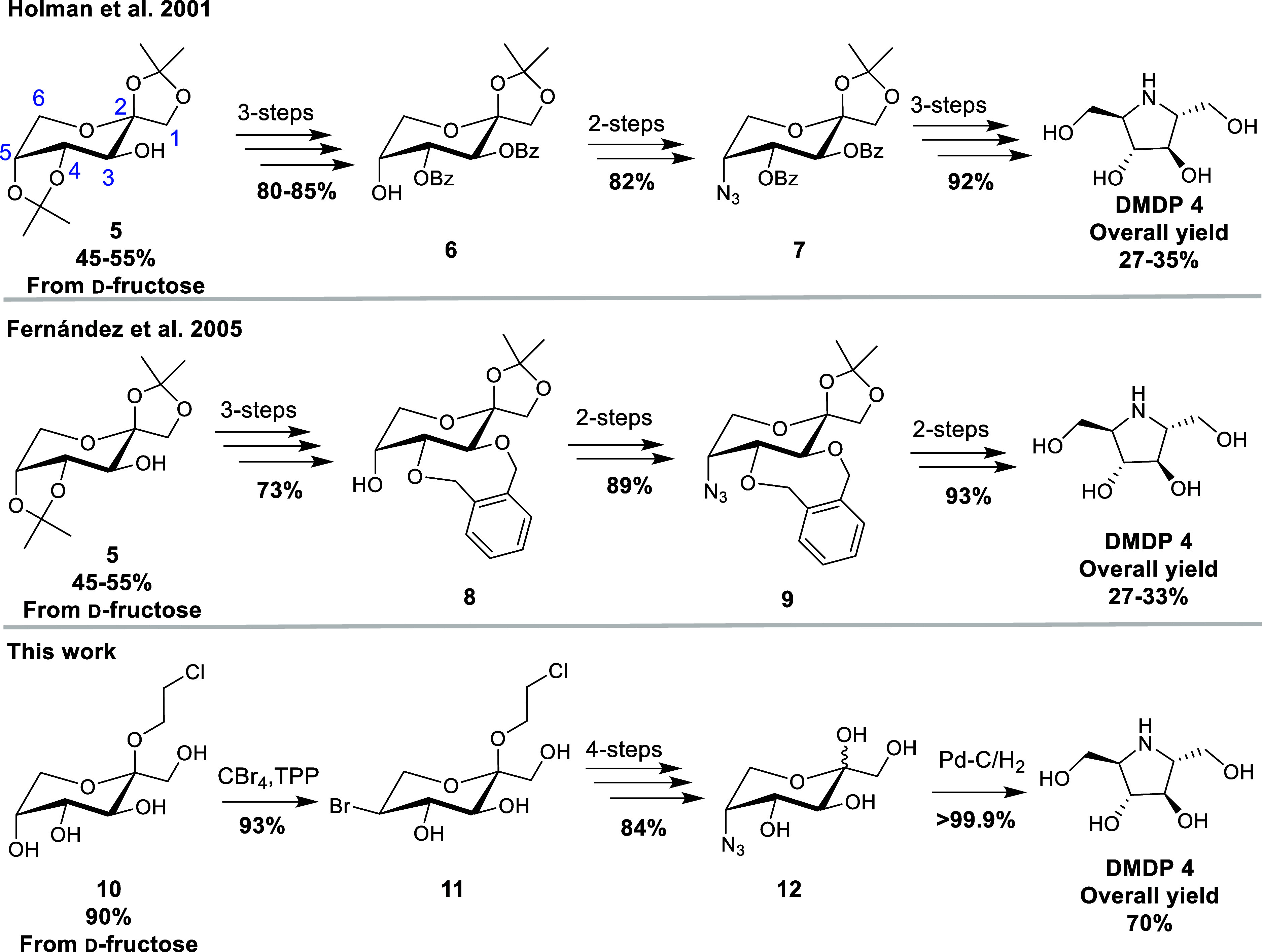
Previous Literature Preparations of DMDP (**4**) from d-Fructose^15−19^ Overall yields are from d-fructose.

DMDP (**4**) and its derivatives have been reported to act as both antiviral (HIV, influenza)^4,5^ and antituberculosis agents,^6,7^ while also functioning as an antifeedant for insects.^8^ This has led to DMDP (**4**) being a synthetic target of interest for organic chemists from a variety of starting materials, including carbohydrates such as l-xylose.^9−14^ Most often its chemical synthesis starts from d-fructose (Scheme 1),^15−19^ which after regioselective C-5 double inversion with a nitrogen nucleophile, affords an intermediate which undergoes reductive amination to afford **4** stereoselectively. Despite this, DMDP (**4**) remains prohibitively expensive from commercial sources, with 1 g costing $10,000 to 60,000 USD, making exploration of the scaffold difficult.

Previous literature preparations of DMDP (**4**) from d-fructose are significantly limited in yield by a necessity to start from the kinetic diacetonide, 1,2:4,5-di-*O*-isopropylidene-β-d-fructopyranose (**5**), which is only available from d-fructose in a 45–55% yield.^20^ This material then requires several selective protecting group manipulations at O-3/O-4 to selectively introduce nitrogen at C-5,^15−19^ resulting in long multistep syntheses that require multiple rounds of chromatography (Scheme 1).

Recently, we reported a practical large-scale preparation of the valuable C-2 epimer of 1-DNJ (**1**), 1-deoxymannojirimycin (1,5-dideoxy-1,6-imino-d-mannitol, 1-DMJ) from d-fructose using the Mitsunobu reaction.^21^ Continuing our interest in developing practical synthetic methods suitable for the large-scale preparation of iminosugars derived from d-fructose, we set out to develop a novel synthetic strategy to prepare DMDP (**4**).

## Results and Discussion

Given the shortcomings of the diacetonide pathways, we decided to use 2-chloroethyl β-d-fructopyranoside (**10**) as a starting material^22^ since **10** can be prepared in a single step from d-fructose via a Fischer glycosylation (1.3% AcCl in 2-chloroethanol) in 90% yield as a white crystalline solid, which readily precipitates from the reaction mixture via a facile dynamic crystallization process.^23^ Although **10** is commercially available, emphasis should be placed on the simplicity of its preparation, given only a handful of publications have utilized this material, which is typically used for the preparation of spirocyclic derivatives.^23−28^

In the original report by Chan et al.^22^ a regioselective Appel reaction [CCl_4_/triphenylphosphine (TPP)] was reported,^29^ to prepare 2-chloroethyl 5-chloro-5-deoxy-α-l-sorbopyranoside from **10** in 85% yield. A similar regioselective C-5 bromination using CBr_4_/TPP has also been reported on methyl and octyl β-d-fructopyranoside in 92% and 83% yield, respectively.^30,31^

Adapting these literature reports, we chose to utilize CBr_4_/TPP due to the hazards associated with CCl_4_.^32^ Additionally, the C-5 bromide intermediate would be a better electrophile in the follow-up transformation while also being more stable than the equivalent C-5 iodide, which has been reported to eliminate on related substrates.^7,31,33^

**Scheme 2:**
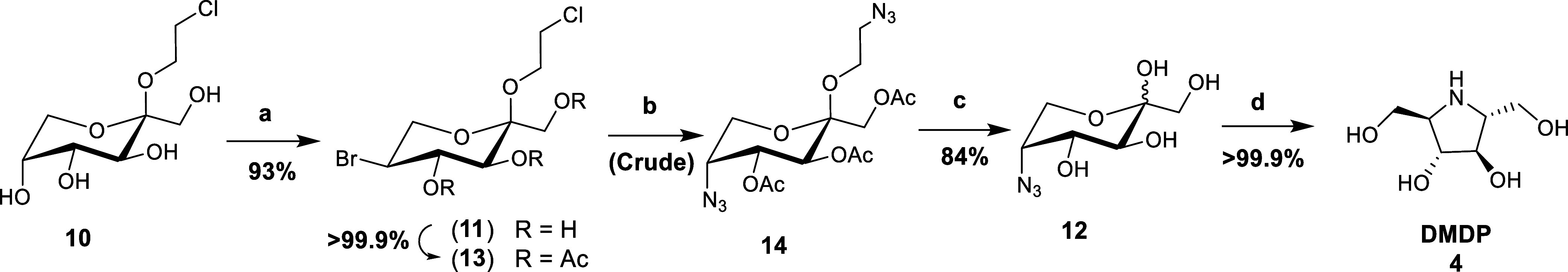
Synthesis of DMDP(**4**) from 2-Chloroethyl β-d-Fructopyranoside (**10**) Reagents and conditions: (a) (i) CBr_4_, TPP, pyridine, 0 to 80 °C 2 h; (ii) Ac_2_O, pyridine O/N; (b) NaN_3_, DMSO, 100 °C O/N; (c) (i) NaOMe, MeOH O/N; (ii) 90% aq. TFA 1.5 h; and (d) Pd/C–H_2_, 1:1 H_2_O:MeOH, 4 bar, O/N.

Using CBr_4_/TPP, we observed a 93% yield of the desired bromide **11** (Scheme 2). Alternative triphenylphosphine-based halogenation reagents such as TPP/NBS, I_2_/TPP/Imidazole, TPPCl_2_, and TPPBr_2_ were also assessed for this step; however, complex mixtures of products were consistently observed. It is possible that expeditious water allows for the formation of furanoside byproducts via a tertiary fructopyranosyl carbocation intermediate. We have previously reported a similar process to occur under even mildly acidic conditions in ketoses such as d-fructose.^34^ Notably, the bromide **11** at this stage could be easily extracted into the aq. phase, facilitating the practical removal of TPP, TPPO, and CHBr_3_ prior to chromatography.

Since bromide **11** contains two halogens, we planned on displacing both the primary chloride and the secondary bromide using NaN_3_. Surprisingly, attempts to convert bromide **11** into the corresponding 2-azidoethyl 5-azido-5-deoxy-α-l-sorbopyranoside using NaN_3_ in DMF were initially unsuccessful, with the major product being 2-azidoethyl 5-bromo-5-deoxy-α-l-sorbopyranoside. Similar issues have been previously reported during the preparation of the octyl equivalent,^31^ thus we decided to acetylate the partially protected bromide to afford **13** and switch to NaN_3_ in DMSO, which resulted in near-quantitative conversion to the diazido derivative **14**.

Due to the high yields of **14** and similar *R*_f_ to the bromide **13**, we found it most practical to remove the acetyl groups using NaOMe in MeOH, followed by aq. hydrolysis using 9:1 TFA/H_2_O to remove the 2-azido-ethyl protecting group to afford 5-azido-5-deoxy-d-fructopyranoside **12** as a 5:95(α/β) mixture of anomers. To avoid the potential risk of handling concentrated 2-azidoethanol, which is a potentially explosive material [(*N*_C_ + *N*_O_)/*N*_N_ ≥ 3) *N* = number of atoms],^35,36^ the aq. solution was washed with CHCl_3_ prior to chromatography, allowing for safe purification of the aq. soluble azide **12**.

**Scheme 3:**
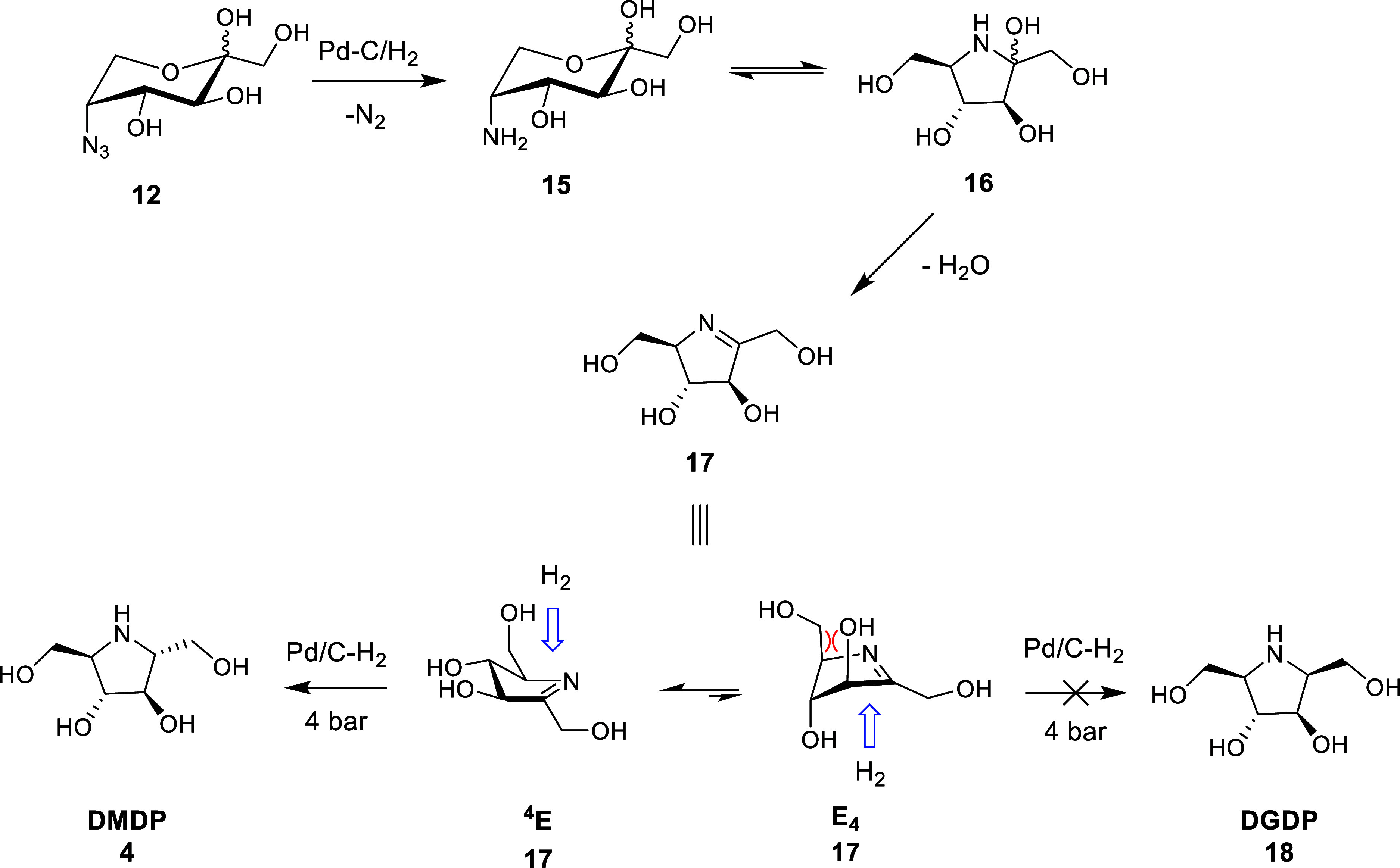
Stereoselective Reduction of the Imine **17** Formed During the Reductive Amination of 5-Azido-5-deoxy-d-fructopyranose (**12**) Inside attack of a small nucleophile (H_2_) proceeds from the same face as the out-of-plane carbon since this forms a lower-energy staggered (inside) vs eclipsed (outside) product.^37−39^

Under literature conditions,^16^ azide **12** was reduced at 4 bar in a Parr apparatus using Pd/C–H_2_ in 1:1 MeOH/H_2_O to afford the C_2_ symmetric product DMDP (**4**) on a 5 g scale in an overall yield of 70% over 7 steps (with 2 purifications) from d-fructose. Notably, the overall yield doubles those previously reported from d-fructose (Scheme 1). The reduction of azide **12** to form **4** was stereoselective and quantitative, with the product forming via inside attack of H_2_ from the top face (re) in the ^4^E conformer (Scheme 3).^37−39^ We hypothesize that stereoselectivity is driven by unfavorable pseudo-1,3-diaxial interactions in the E_4_ conformer, which disfavor conformational pseudorotation into this conformer at elevated pressure, although it should be noted that at atmospheric pressure diminished stereocontrol was observed [d.r 90:10 DMDP (**4**)/DGDP (2,5-dideoxy-2,5-imino-d-glucitol), **18**].

With the successful preparation of DMDP (**4**), we envisioned that the regioselective Appel reaction may also work on the three remaining ketopyranoses in a ^2^C_5_ conformation, therefore allowing for access to valuable building blocks suitable for pyrrolidine iminosugar synthesis. Pyrrolidine iminosugars have been previously synthesized from protected versions of the ketopyranoses 5-azido-5-deoxy-l-sorbo-, 5-azido-5-deoxy-d-psico-, and 5-azido-5-deoxy-l-tagatopyranose, affording DGDP (**18**), 2,5-dideoxy-2,5-imino-d-altritol (DIA), and 2,5-dideoxy-2,5-imino-d-galactitol (DGADP), respectively.^15,40,41^

Although outside of our initial scope, we were curious to prepare DGDP (**18**), the C-2 diastereomer of DMDP (**4**) since there are conflicting literature reports for the product of the reductive amination of 5-azido-5-deoxy-α,β-l-sorbopyranose. Although one report indicated that the reduction was stereoselective and formed exclusively DGDP (**18**),^42^ most other authors report a 90:10 diastereomeric ratio of DGDP (**18**) to DMDP (**4**) when the reduction is run at 4 bar under Pd/C–H_2_ catalysis.^43,44^

**Scheme 4:**
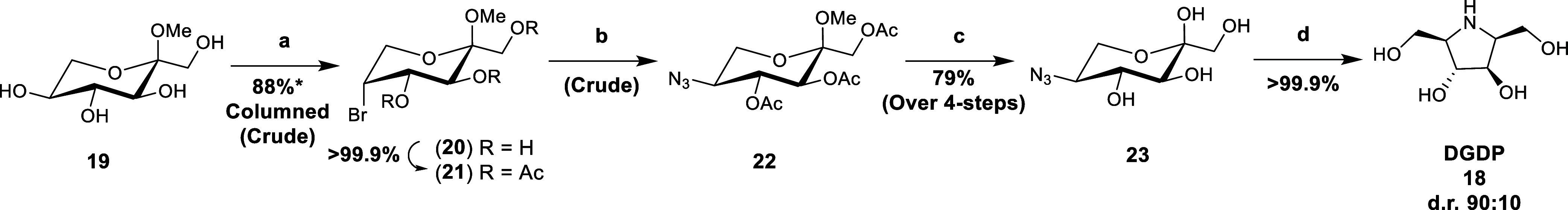
Synthesis of DGDP (**18**) from Methyl α-l-Sorbopyranoside (**19**) Reagents and conditions: (a) (i) CBr_4_, TPP, pyridine 0 °C to 80 °C 2 h; (ii) Ac_2_O, pyridine O/N; (b) NaN_3_, DMSO, 100 °C O/N; (c) (i) NaOMe, MeOH O/N; (ii) 90% aq. TFA 1.5 h; and (d) Pd/C–H_2_, 1:1 H_2_O/MeOH, 4 bar, O/N.

Via an identical process to the DMDP (**4**) synthesis, albeit starting from the crystalline starting material methyl α-l-sorbopyranoside (**19**) (81% from l-sorbose),^45^ we prepared the aq. soluble bromide (**20**) (Scheme 4). There was a trace of an uncharacterized, identical *R*_f_ inseparable impurity we postulate to be the C-3 bromide. This material was acetylated and converted into azide **22**, which was deprotected and purified as the unprotected derivative 5-azido-5-deoxy-α-l-sorbopyranose (**23**), which was consistent with previous literature preparations and was easily separable from the aforementioned impurity via flash chromatography.^46^

**Scheme 5:**
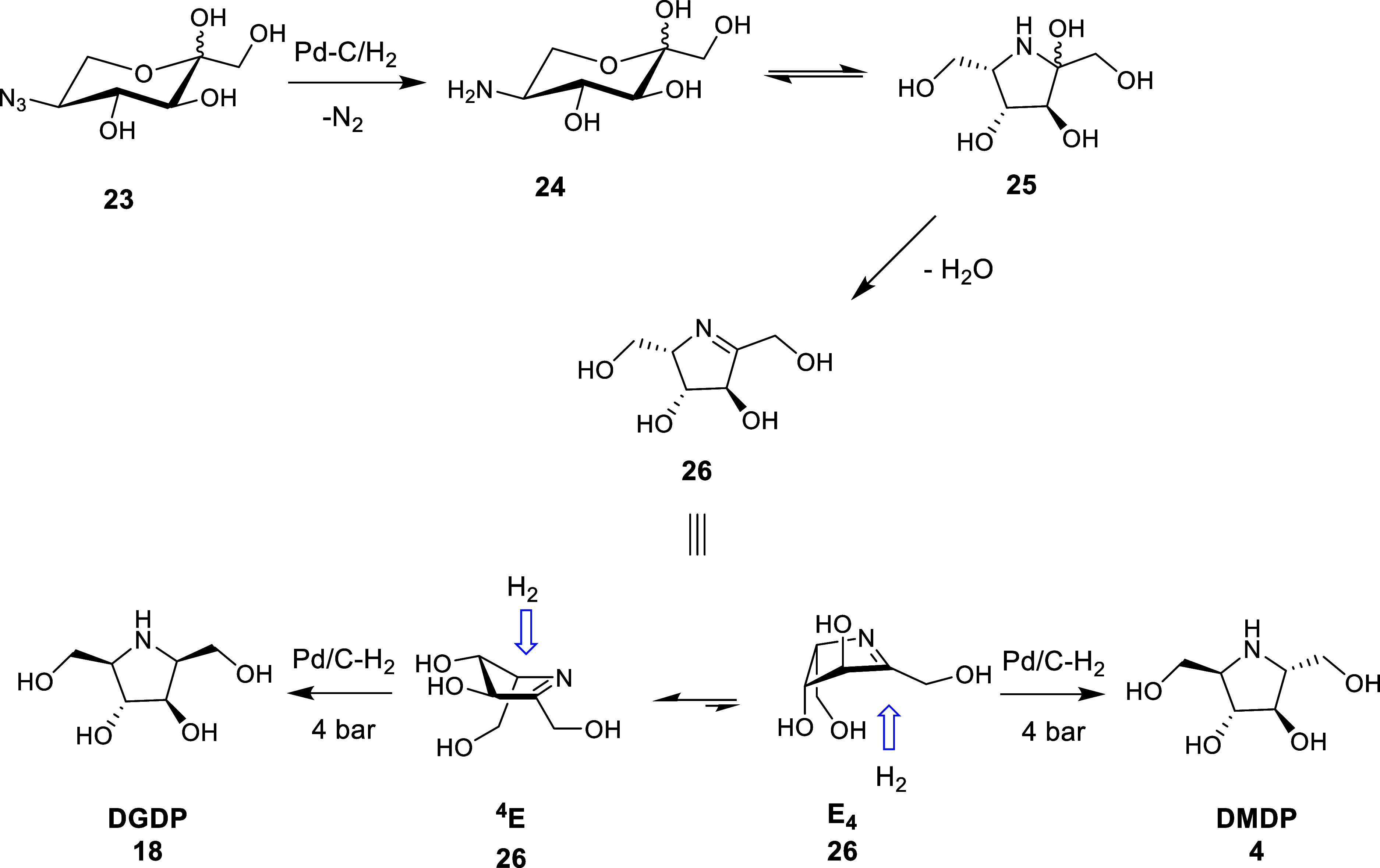
Stereoselective Reduction of the Imine formed During the Reductive Amination of 5-Azido-5-deoxy-l-sorbopyranose (**23**) Conformational pseudo-rotation allows reduction to occur on both envelope conformers.

In our hands, we consistently observed a 90:10 diastereomeric ratio of DGDP (**18**) to DMDP (**4**), which is consistent with other literature reports.^43,44^ We hypothesize that the lack of a disfavorable 1,3 pseudodiaxial interaction in the E_4_ conformer results in an inside attack from the undesired *re* face, resulting in the formation of DMDP (**4**) (Scheme 5). Consistent with our results for DMDP (**4**), when the reduction was undertaken at atmospheric pressure, we observed an 80:20 diastereomeric ratio of DGDP (**18**) to DMDP (**4**), clearly indicating that hydrogenation pressure plays an important role in the face selectivity of the reduction of cyclic imines, presumably by shifting the conformational equilibrium.^44,47^

Notably, literature reports that improved stereocontrol (d.r. 98:2) can be achieved using a rhodium catalyst (Rh/alumina);^44^ however, we did not explore this further. Additionally, conformationally restrictive protecting groups have been used to thermodynamically disfavor conformational pseudorotation into the E_4_ conformer due to 8-membered ring strain.^15,48^

**Scheme 6:**
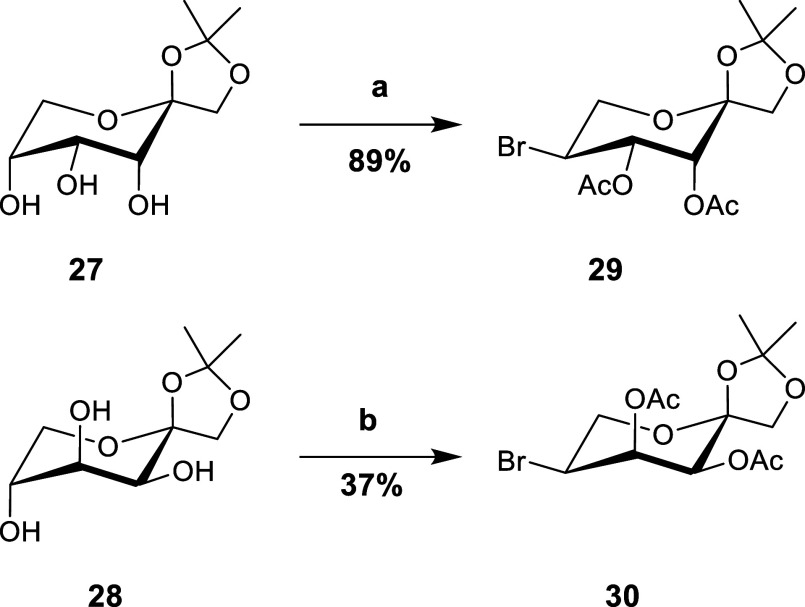
Synthesis of the 5-Bromides of 1,2-*O*-isopropylidene-β-d-psicopyranose (**27**) and 1,2-*O*-isopropylidene-β-d-tagatopyranose (**28**) Reagents and conditions: (a) (i) CBr_4_, TPP, pyridine 0 °C to 80 °C 2 h; (ii) Ac_2_O, pyridine O/N; (b) (i) CBr_4_, TPP, pyridine 0 °C to 80 °C 24 h; and (ii) Ac_2_O, pyridine O/N.

Eager to further probe the regioselectivity of the Appel reaction on the remaining ketopyranosides in a ^2^C_5_ conformation, we prepared both 1,2-*O*-isopropylidene-β-d-psicopyranose (**27**) and 1,2-*O*-isopropylidene-β-d-tagatopyranose (**28**) using literature procedures from d-fructose.^49−51^ Both of these reacted regioselectively at C-5 to afford **29** and **30** in 89% and 37% yields, respectively (Scheme 6). Confirmation of stereocenter inversion was determined by comparing ^3^J_H-3-H-4_ and ^3^J_H-4-H-5_ coupling constants in combination with the distinct upfield shift observed for C-5 in the^13^ C NMR spectrum. It was surprising that **28** resisted nucleophilic attack to afford only a 37% yield of **30** after 24 h at reflux. Based on these results, we believe the regioselectivity is driven by a combination of electronic and steric effects hindering reactivity at C3/C-4 in the ^2^C_5_ conformation.

In summary, using a regioselective Appel reaction, we have developed two high-yielding multigram suitable methods to access the valuable iminosugars DMDP (**4**) and DGDP (**18**) from d-fructose and l-sorbose in 70% and 56% overall yields, respectively, with the latter in diminished diastereomeric purity (d.r. 90:10). These processes are highly practical, utilizing aqueous extraction to facilitate the practical removal of Appel reaction byproducts, making the reported syntheses highly suited to the large-scale preparation of iminosugars. We also report for the first time the application of a regioselective Appel reaction to prepare the epimeric C-5 bromide on selectively protected d-psico- and d-tagatopyranose scaffolds in a ^2^C_5_ conformation. These bromides will allow for improved access to the C-5 derivatives of ketopyranoses for the synthesis of valuable synthetic targets such as iminosugars or 5-thiosugars. We are currently working toward the generation of libraries of DMDP and DGDP derivatives to identify more potent glycosidase inhibitors for therapeutic purposes.^52^

## Experimental Section

^1^H and ^13^C spectra were recorded on a Brüker Avance 400 MHz spectrometer. Chemical shifts are in parts per million relative to the solvent used (CDCl_3_: 7.26 for ^1^H and 77.16 for ^13^C, D_2_O: 4.79 for ^1^H). Low-resolution electrospray ionization (ESI) mass spectra were recorded using a Shimadzu LCMS-2020 single quad UPLC (ESI-MS). High-resolution mass spectra were recorded using a maXis II ETD ESI quadrupole time-of-flight (QTOF) or Agilent 6530 QTOF mass spectrometer. Melting points were measured using open capillary tubes on a Gallenkamp MPD350.BM3.5 melting point apparatus and are uncorrected. Optical rotations were recorded on a Bellingham + Stanley ADP430 instrument (specific rotation, tube length: 50 mm, concentrations in g/100 mL). Thin-layer chromatography was run on a Merck 60 F254 aluminum-backed silica plates. Reactions run at elevated temperatures were heated in a heating mantle. Carbohydrates were identified via an ethanolic 5% sulfuric acid stain, and UV-active compounds were visualized under a short-wave (254 nm) light source. Azides were converted to amines via dipping the TLC plate into CH_2_Cl_2_ containing triphenylphosphine, followed by staining with ninhydrin. Column chromatography was performed using Davisil 40–63 μm silica gel using distilled solvents. Reagents were used as received, and all reaction solvents used were of anhydrous high-performance liquid chromatography grade, unless stated otherwise. Structural assignments were made using additional information from gCOSY, gHSQC, and gHMBC experiments.
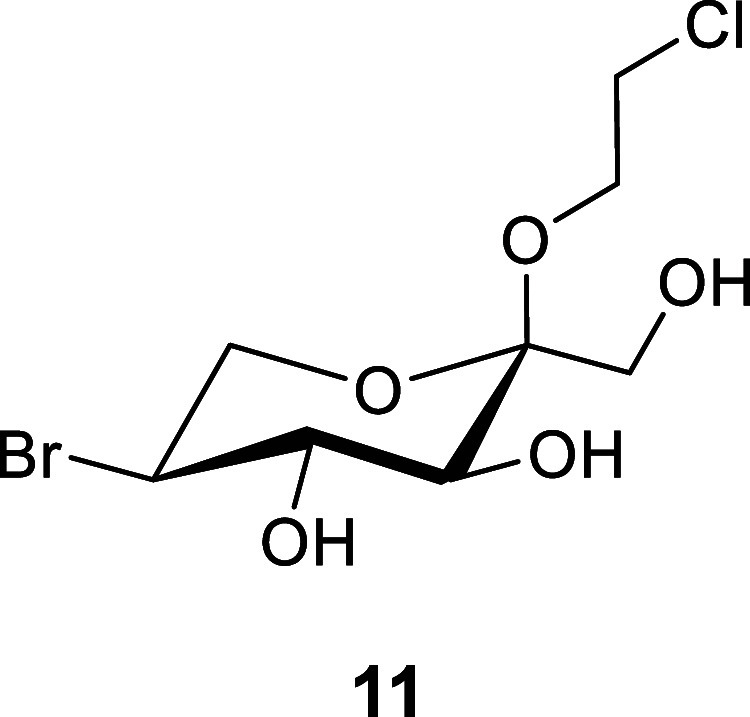


### 2-Chloroethyl 5-Bromo-5-deoxy-α-l-sorbopyranoside (**11**)

To a round-bottom flask containing 2-chloroethyl-β-d-fructopyranoside **10**(22) (10.0 g, 41.2 mmol) was added triphenylphosphine (43.2 g, 164.8 mmol, 4.0 equiv). The solids were dissolved in pyridine (125 mL), and the reaction vessel was placed in an ice bath and allowed to cool. Once cool, CBr_4_ (27.3 g, 82.4 mmol, 2.0 equiv) was added in several portions (3–5 g) and allowed to stir for 10 min before being heated to 80 °C. After 2 h at this temperature, TLC indicated consumption of the starting material to a higher spot (*R*_f_ = 0.7, 8:2, DCM/MeOH). The reaction vessel was allowed to cool to rt before an excess of methanol (30 mL) was added. The solution was stirred for 15 min before being concentrated in vacuo to afford a dark syrup. The solution was azeotropically dried by concentration in vacuo 3 times with a 1:1 toluene/MeOH (150 mL) solution to dryness. The syrup was then dissolved in 1:1 DCM/H_2_O (2.0 L), and the organic phase was extracted 3 times with H_2_O (1.0 L). The aqueous phases were combined and concentrated in vacuo to afford a dark syrup, which was purified via column chromatography [DCM to 95:5, DCM/MeOH] to afford 2-chloroethyl 5-bromo-5-deoxy-α-l-sorbopyranoside (**11**) as a colorless syrup (11.7 g, 38.3 mmol, 93%), which foamed upon extended drying and was used immediately.

^1^H NMR (400 MHz, MeOD):δ 4.00–3.91 (m, 1H), 3.90–3.59 (m, 10H), 3.61–3.50 (m, 1H).

^13^C NMR (101 MHz, MeOD):δ 99.9 (C-2), 73.3 (C-3), 72.5 (C-4), 62.8 (C-6), 60.8(C-1), 60.7 (C-1′), 49.0 (C-5), 42.2 (C-2′).

HRMS(ESI/Q-TOF) *m*/*z*: [M + Na]+ calcd for C_8_H_14_^79^BrClNaO_5_ 326.9615; Found, 326.9605.

### 2-Chloroethyl 1,3,4-Tri-*O*-acetyl-5-bromo-5-deoxy-α-l-sorbopyranoside (**13**)

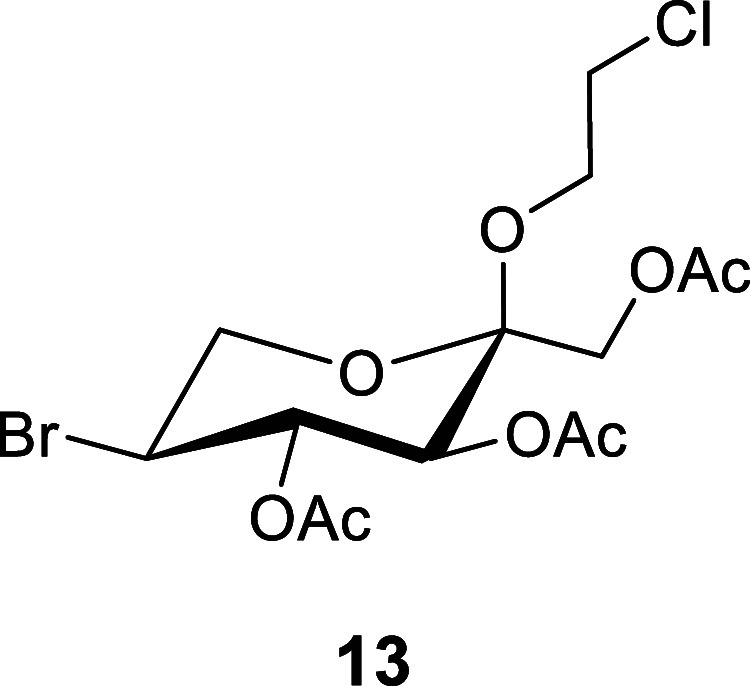



The foam was dissolved in pyridine (60 mL) and Ac_2_O (20 mL, 211 mmol), and the mixture was stirred in the mixture of O/N at rt. Following this, a single higher *R*_f_ spot on TLC (*R*_f_ = 0.3, 2:1, hexanes/EtOAc) was observed, and the reaction vessel was cooled in an ice bath before methanol (30 mL) was slowly added to degrade excess Ac_2_O. The solution was concentrated in vacuo and azeotroped with toluene (100 mL) 3 times to afford the title compound (16.57 g, 38.39 mmol, >99.9%) as a white amorphous solid.

^1^H NMR (400 MHz, CDCl_3_): δ 5.51 (t, *J* = 9.7 Hz, H-4, 1H), 5.12 (d, *J* = 9.8 Hz, H-3, 1H), 4.24 (d, *J* = 11.8 Hz, H-1a, 1H), 4.08–3.90 (m, H-1b, H-5, H-1′a, H-1′b, 4H), 3.86–3.64 (m, H-6a, H-6b, H-2′a, H-2′b, 4H), 2.07 (s, OAc, 3H), 2.06 (s, OAc, 3H), 2.04 (s, OAc, 3H);

^13^C{^1^H} NMR (101 MHz, CDCl_3_): δ 170.2 (C=O, OAc), 169.9 (C=O, OAc), 169.7 (C=O, OAc), 99.1 (C-2), 72.5 (C-4), 71.1 (C-3), 63.6 (C-1′), 62.4 (C-1), 62.2 (C-6), 44.3 (C-5), 42.4 (C-2′), 20.8 (CH_3_, OAc), 20.8 (CH_3_, OAc), 20.7 (CH_3_, OAc).

HRMS (ESI/Q-TOF) *m*/*z*: [M + Na]^+^ calcd for C_14_H_20_^79^BrClNaO_8_, 452.9922; found, 452.9918.
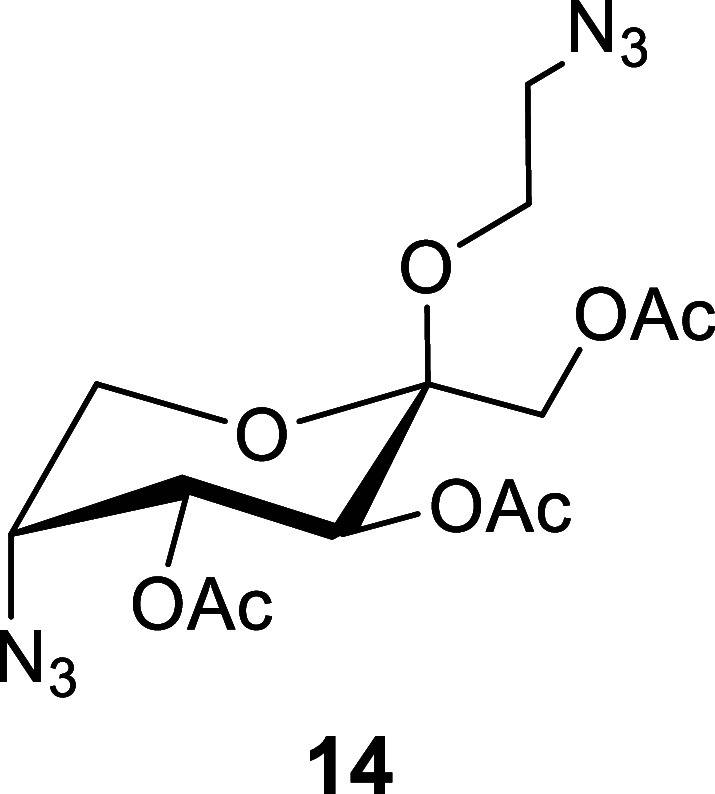


### 2-Azidoethyl 1,3,4-Tri-*O*-acetyl-5-azido-5-deoxy-β-d-fructopyranoside (**14**)

To a round-bottom flask containing 2-chloroethyl 1,3,4-tri-*O*-acetyl-5-bromo-5-deoxy-α-l-sorbopyranoside (**13**) (16.5 g, 38.3 mmol) were added NaN_3_ (12.4 g, 190 mmol, 5.0 equiv) and DMSO (80 mL). The reaction mixture was then heated to 100 °C and stirred overnight. The next day, the reaction vessel was allowed to cool to rt before being diluted with EtOAc (300 mL) and H_2_O (200 mL) and moved into a separatory funnel. The solution was then partitioned, and the organic phase was repeatedly washed with satd. brine before being concentrated in vacuo to afford a crude, slightly slower-running solid (*R*_f_ = 0.3, 2:1, hexanes/EtOAc), which was used in the next reaction immediately crude. An analytical sample of **14** was isolated and purified in this solvent system for characterization purposes.

^1^H NMR (400 MHz, CDCl_3_): δ 5.56 (d, *J* = 10.4 Hz, H-3, 1H), 5.36 (dd, *J* = 10.5, 3.8 Hz, H-4, 1H), 4.25 (d, *J* = 11.9 Hz, H-1a, 1H), 4.17–4.12 (m, H-5, 1H), 4.08 (d, *J* = 11.9 Hz, H-1b, 1H), 3.97 (dd, *J* = 12.7, 1.8 Hz, H-6a, 1H), 3.83 (dd, *J* = 12.6, 1.8 Hz, H-6b, 1H), 3.76–3.60 (m, H-1′a, H-1′b, 2H), 3.48–3.41 (m, H-2′a, H-2′b, 2H), 2.11 (s, OAc, 3H), 2.09 (s, OAc, 3H), 2.07 (s, OAc, 3H).

^13^C{^1^H} NMR (101 MHz, CDCl_3_): δ 170.4(C=O, OAc), 170.4 (C=O, OAc), 170.0 (C=O, OAc), 99.3 (C-2), 70.5 (C-4), 67.3 (C-3), 62.5 (C-1), 61.8 (C-6), 61.2 (C-1′), 59.8 (C-5), 50.7 (C-2′), 20.8 (CH_3_, OAc), 20.8 (CH_3_, OAc), 20.7 (CH_3_, OAc).

HRMS (ESI/Q-TOF) *m*/*z*: [M + Na]+ calcd for C_14_H_20_N_6_NaO_8_ 423.1234; found, 423.1251.
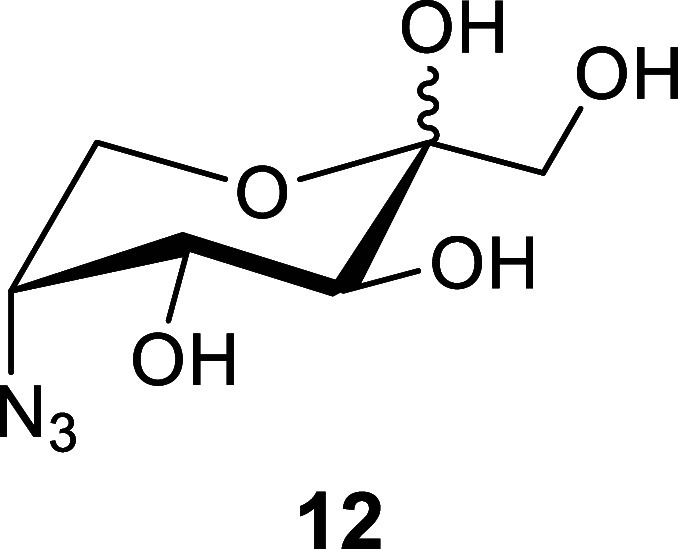


### 5-Azido-5-deoxy-α,β-d-fructopyranose (**12**)

The crude solid **14** from the previous reaction was dissolved in MeOH before a catalytic amount of NaOMe (0.2 g, 3.77 mmol) was added, and the solution was stirred overnight. The next day, the solution was concentrated in vacuo before being dissolved in 90% aq. TFA (100 mL) and stirred at rt. After 1.5 h, TLC indicated complete conversion of the starting material to a lower running spot (*R*_f_ = 0.8 to *R*_f_ = 0.25, DCM/MeOH, 9:1). **Caution:** Acidic hydrolysis forms 2-azidoethanol, which is a potentially explosive material. The aqueous solution was diluted with 200 mL of H_2_O and then repeatedly washed with CHCl_3_ (4 × 100 mL) to remove 2-azidoethanol, which can be decomposed by adding an excess of triphenylphosphine (3–4 equiv) to the organic phase and stirring at rt for >24 h. The aq. phase was concentrated in vacuo before being purified via column chromatography [95:5 to 9:1 to 8:2, DCM/MeOH] to afford **12**(53) as an inseparable mixture of anomers (5:95, α/β) (6.65 g, 32.4 mmol, 84%), which appears as a colorless syrup which crystallized upon extended drying. Some batches we prepared appeared as a colorless/white foam.

^1^H NMR (400 MHz, D_2_O): δ 4.07 (t, *J* = 9.9 Hz, H-4, 1H), 4.03–3.98 (m, H-5, 1H), 3.76 (dd, *J* = 12.8, 1.9 Hz, H-6b, 1H), 3.74 (d, *J* = 9.9 Hz, H-3, 1H), 3.66 (d, *J* = 11.8 Hz, H-1a, H-6a, 2H), 3.50 (d, *J* = 11.7 Hz, H-1b, 1H).

^13^C{^1^H} NMR (101 MHz, D_2_O): δ: 98.1 (C-2β), 97.8 (C-2α), 72.6 (C-4α), 70.3 (C-3α), 69.7 (C-4β), 67.6 (C-3β), 63.5 (C-1β), 63.3(C-1α), 62.5 (C-5β), 61.2 (C-5α), 60.8 (C-6β), 59.9 (C-6α).

HRMS (ESI/Q-TOF) *m*/*z*: [M + Na]+ calcd for C_6_H_11_N_3_NaO_5_ 228.0590; found, 228.0593.

### 2,5-Dideoxy-2,5-imino-d-mannitol (DMDP) (**4**)

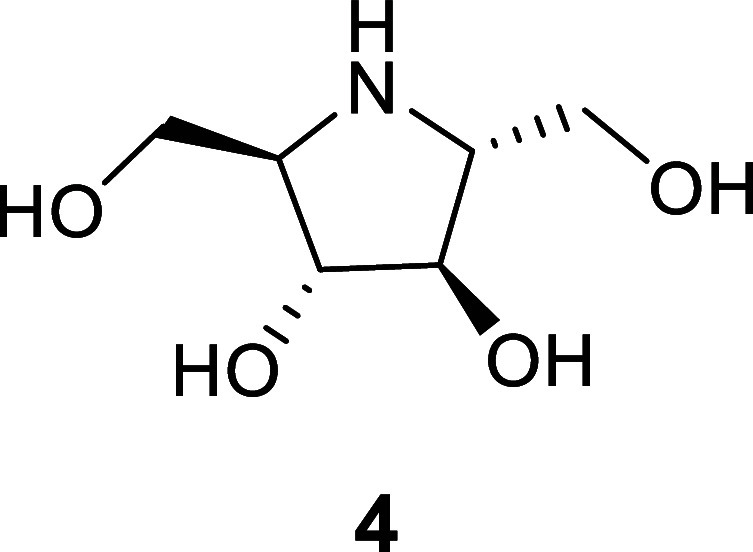



To a round-bottom flask containing 5-azido-5-deoxy-α,β-d-fructopyranose (**12**) (6.65 g, 32.4 mmol, 1.0 equiv), which was evacuated in vacuo and placed under an argon atmosphere, was dissolved in 1:1 H_2_O/MeOH before 10% Pd/C (1 g) was added. The solution was then transferred to a Parr apparatus and reacted under a H_2_ atmosphere at 4 bar for 24 h. After 24 h, the reaction mixture was filtered through diatomaceous earth, and the catalyst was repeatedly washed with MeOH to afford a colorless filtrate. The solution was concentrated in vacuo and concentrated with methanol (50 mL) three times to afford DMDP (**4**)^53^ (5.29 g, 32.4 mmol, >99.9%) as a colorless solid.

^1^H NMR (400 MHz, D_2_O): δ 3.83 (dd, *J* = 5.6, 2.3 Hz, H-3, H-4, 2H), 3.71 (dd, *J* = 11.7, 4.2 Hz, H-1a, H-6a 2H), 3.62 (dd, *J* = 11.7, 6.3 Hz, H-1b, H-6b, 2H), 3.04 (tdd, *J* = 6.2, 4.3, 2.3 Hz, H-2, H-5 2H).

^13^C{^1^H} NMR (101 MHz, D_2_O): δ: 77.7 (C-3, C-4), 61.9 (C-1, C-6), 61.7 (C-2, C-4).

MP: 115–117 °C (lit 115–117 °C).^53^

[α]_D_^25.0^ = +59.9 (*c* 1.0 in H_2_O) (Lit. [α]_D_ = +55.8) (*c* 1.0 in H_2_O)^53^

HRMS (ESI/Q-TOF) *m*/*z*: [M + H]+ calcd for C_6_H_14_NO_4_ 164.0917; found, 164.0917.

### Methyl 1,3,4-Tri-*O*-acetyl-5-bromo-5-deoxy-β-d-fructopyranoside (**21**)

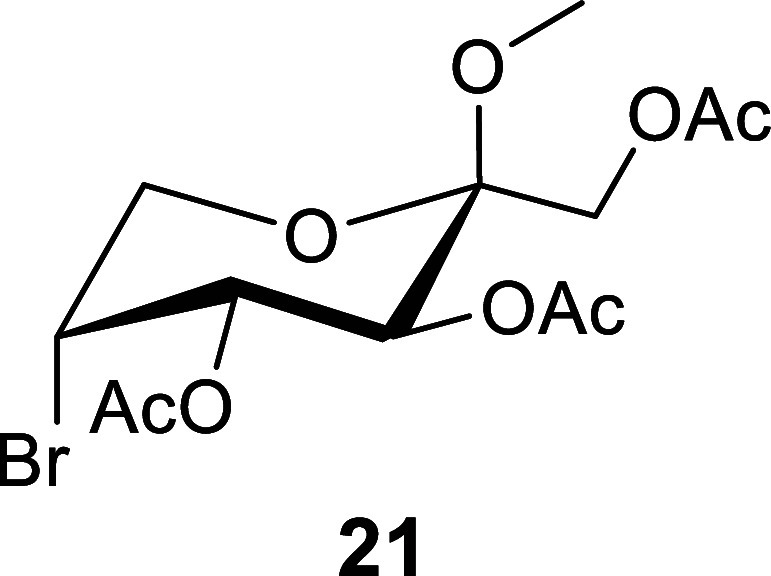



To a round-bottom flask containing methyl-α-l-sorbopyranoside (**19**)^45^ (8 g, 41.2 mmol, 1.0 equiv) was added triphenylphosphine (43.2 g, 164.8 mmol, 4 equiv). The solids were dissolved in pyridine (125 mL), and the reaction vessel was placed in an ice bath and allowed to cool. Once cool, CBr_4_ (27.3 g, 82.4 mmol, 2.0 equiv) was added in several portions (3–5 g) and allowed to stir for 10 min before being heated to 80 °C. After 2 h at this temperature, TLC indicated consumption of the starting material to a higher Rf spot (*R*_f_ = 0.7, 8:2, DCM/MeOH). The reaction vessel was allowed to cool to rt before an excess of methanol was added. The solution was allowed to stir for 15 min before being concentrated in vacuo to afford a dark syrup. The solution was azeotropically dried by concentration in vacuo 3 times with a 1:1 toluene/MeOH (150 mL) solution to dryness. The syrup was then dissolved in 1:1 DCM/H_2_O (2.0 L), and the organic phase was extracted 3 times with H_2_O (1.0 L). The organic phase was extracted 3 times with H_2_O (1 L). The aqueous phases were combined and concentrated in vacuo to afford a dark syrup, which was purified via column chromatography [DCM to 95:5, DCM/MeOH] to afford methyl 5-bromo-5-deoxy-β-d-fructopyranoside (**20**) as a colorless syrup (9.33 g, 36.3 mmol, 88%), which foamed on extended drying. The foam was dissolved in pyridine (60 mL) and Ac_2_O (20 mL, 211 mmol) and stirred overnight at rt. The next day the reaction vessel was cooled in an ice bath before methanol (30 mL) was slowly added to degrade excess Ac_2_O. The solution was azeotropically dried by concentration in vacuo with toluene (150 mL) to afford the title compound (15.6 g, 36.3 mmol, >99.9%) as an off-white amorphous solid.

^1^H NMR (400 MHz, CDCl_3_): δ 5.59 (d, *J* = 10.3 Hz, H-3, 1H), 5.00 (dd, *J* = 10.3, 3.9 Hz, H-4, 1H), 4.59–4.53 (m, H-5, 1H), 4.17 (d, *J* = 11.9 Hz, H-1a, 1H), 4.10 (d, *J* = 11.9 Hz, H-1b, 1H), 4.08 (dd, *J* = 13.2, 1.9 Hz, H-6a, 1H), 3.91 (dd, *J* = 13.3, 1.7 Hz, H-6b, 1H), 3.30 (s, OMe, 3H), 2.06 (s, OAc, 3H), 2.03 (s, OAc, 3H), 2.02 (s, OAc, 3H).

^13^C{^1^H} NMR (101 MHz, CDCl_3_): δ 170.3 (C=O, OAc), 170.2 (C=O, OAc), 169.6 (C=O, OAc), 99.2(C-2), 69.1 (C-4), 67.8 (C-3), 64.0 (C-1), 62.0 (C-6) 50.1 (C-5), 49.2 (OMe), 20.8 (CH_3_, OAc), 20.7 (CH_3_, OAc), 20.7 (CH_3_, OAc).

MS (ESI) *m*/*z*: [M + Na]+ calcd for C_13_H_19_BrNaO_8_ 406.13; found, 405.0 and 407.0.

### Methyl 1,3,4-Tri-*O*-acetyl-5-azido-5-deoxy-α-l-sorbopyranoside (**22**)

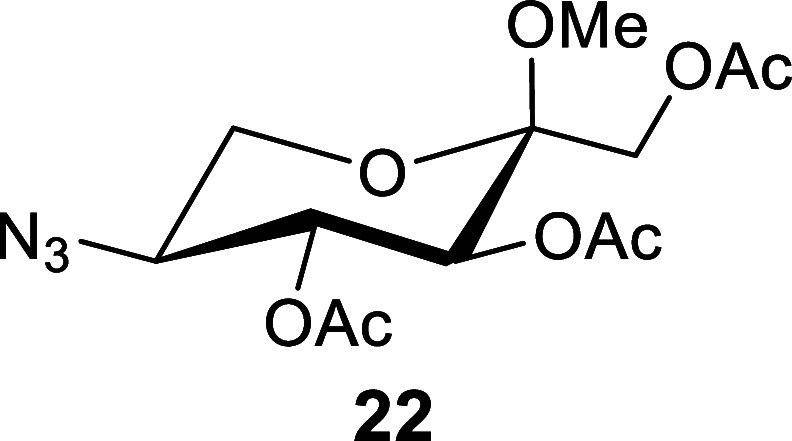



To a round-bottom flask containing slightly crude **21** (15.6 g, 36.3 mmol) was added NaN_3_ (11.8 g, 181 mmol, 5 equiv) and DMSO (80 mL). The reaction mixture was then heated to 100 °C and stirred overnight. The next day, the reaction vessel was allowed to cool and to rt before being diluted with EtOAc (300 mL) and H_2_O (300 mL). The solution was then partitioned, and the organic phase repeatedly washed with satd. brine before being concentrated in vacuo to afford a crude, slightly slower-running solid (*R*_f_ 0.3, 2:1, Hexanes/EtOAc), which was used in the next reaction crude. An analytical sample of **22** was isolated and purified in this solvent system for characterization purposes.

^1^H NMR (400 MHz, CDCl_3_): δ 5.35 (t, *J* = 9.8 Hz, H-4, 1H), 5.11 (d, *J* = 10.0 Hz, H-3, 1H), 4.20 (d, *J* = 11.8 Hz, H-1a, 1H), 4.04 (d, *J* = 11.8 Hz, H-1b, 1H), 3.82 (dd, *J* = 11.1, 5.9 Hz, H-6a, 1H), 3.71 (ddd, *J* = 11.2, 9.7, 5.8 Hz, H-5, 1H), 3.41 (t, *J* = 11.2 Hz, H-6b 1H), 3.29 (s, OMe, 3H), 2.04 (s, OAc, 3H), 2.04 (s, OAc, 3H), 2.02 (s, OAc, 3H).

^13^C{^1^H} NMR (101 MHz, CDCl_3_): δ 170.2 (C=O, OAc), 170.0 (C=O, OAc), 169.9 (C=O, OAc), 98.7 (C-2), 71.8 (C-4), 70.2 (C-3), 61.9 (C-1), 60.7 (C-6), 59.4 (C-5), 49.1 (OMe), 20.7 (CH_3_, OAc), 20.7, (CH_3_, OAc) 20.7 (CH_3_, OAc).

HRMS(ESI/Q-TOF) *m*/*z*: [M + Na]+ calcd for C_13_H_19_N_3_NaO_8_ 368.1064; found, 368.1078.

### 5-Azido-5-deoxy-α-l-sorbopyranose (**23**)

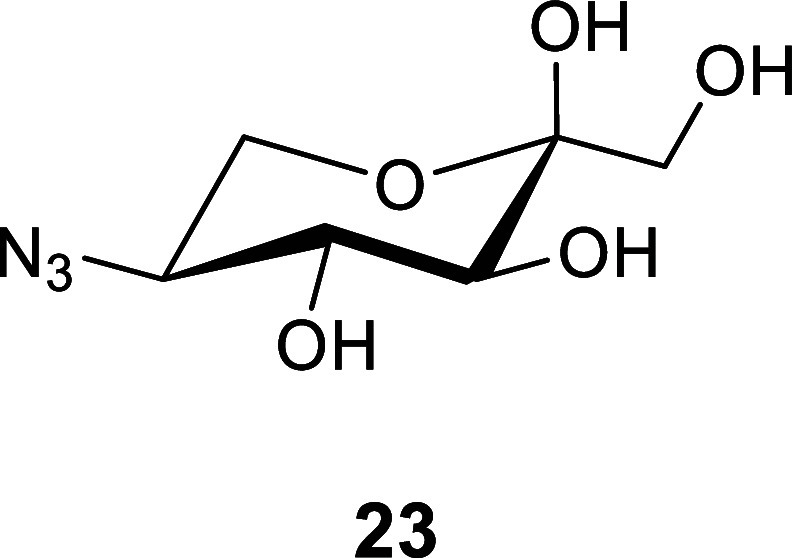



The crude solid **22** from the previous reaction was dissolved in MeOH before a catalytic amount of NaOMe (0.2 g, 3.77 mmol) was added, and the mixture was stirred overnight. The next day, the solution was concentrated in vacuo before the crude solid (*R*_f_ 0.8, 9:1, DCM/MeOH) was dissolved in 90% aq. TFA and stirred at rt. After 1.5 h, TLC indicated complete conversion of the starting material to a slower-moving spot (*R*_f_ = 0.3, 9:1, DCM/MeOH). The solution was then concentrated in vacuo before being purified via column chromatography [95:5 to 9:1 to 8:2, DCM/MeOH] to afford the title compound^54^ (5.90 g, 28.7 mmol, 79%) as a colorless syrup, which crystallized upon extended drying.

^1^H NMR (400 MHz, D_2_O): δ 3.79 (dd, *J* = 10.7, 5.1 Hz, H-6a, 1H), 3.73 (d, *J* = 9.4 Hz, H-4 1H), 3.69 (d, *J* = 5.7 Hz, H-1a, 1H), 3.64 (d, *J* = 10.2 Hz, OH, 1H), 3.59 (d, *J* = 10.8 Hz, H-6b, 1H), 3.56–3.50 (m, H-5, 1H), 3.52 (d, *J* = 9.5 Hz, H-3, 1H), 3.47 (d, *J* = 11.8 Hz, H-1b, 1H).

^13^C{^1^H} NMR (101 MHz, D_2_O): δ: 98.9 (C-2), 73.7 (C-4), 71.4 (C-3), 64.4 (C-1), 62.3 (C-5), 61.0 (C-6).

HRMS(ESI/Q-TOF) *m*/*z*: [M + Na]+ calcd for C_6_H_11_N_3_NaO_5_ 228.0590; found, 228.0597.

### 2,5-Dideoxy-2,5-imino-d-glucitol (DGDP) (**18**)

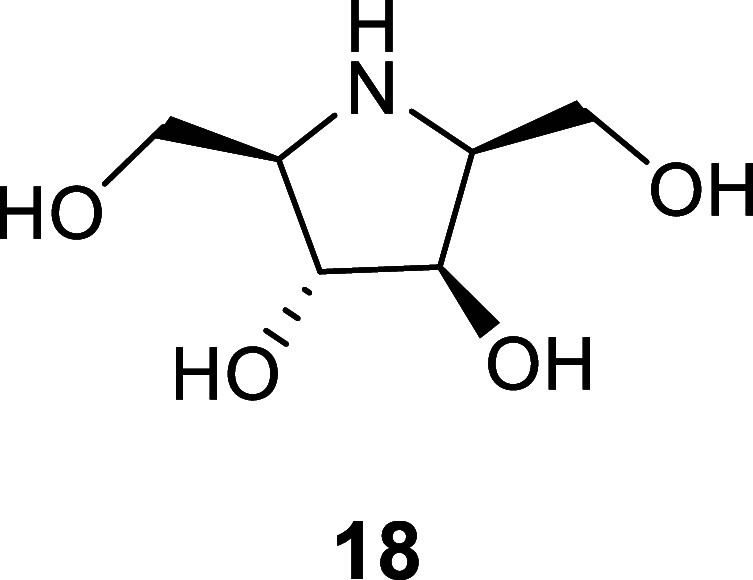



To a round-bottom flask containing 5-azido-5-deoxy-α-l-sorbopyranose (**23**) (5.90 g, 28.7 mmol), which was evacuated in vacuo and placed under an argon atmosphere, was dissolved in 1:1 H_2_O/MeOH before 10% Pd/C (1 g) was added. The solution was then transferred to a Parr apparatus and reacted under a H_2_ atmosphere at 4 bar for 24 h. After 24 h, the reaction mixture was filtered through diatomaceous earth, and the catalyst was repeatedly washed with MeOH to afford a colorless filtrate. The solution was concentrated in vacuo and concentrated with methanol (50 mL) three times to afford DGDP (**18**)^46^ (4.69 g, 28.7 mmol, >99.9%) as a colorless solid as a 9:1 mixture of DGDP/DMDP by ^1^H NMR.

^1^H NMR (400 MHz, D_2_O): δ 4.07 (dd, *J* = 5.2, 2.9 Hz, H-4 1H), 3.82 (dd, *J* = 5.4, 2.9 Hz, H-3, 1H), 3.79–3.57 (m, H-1a, H-1b, H-6a, H-6b, 4H), 3.26 (q, *J* = 6.1 Hz, H-5, 1H), 2.96 (dt, *J* = 6.2, 5.1 Hz, H-2 1H).

^13^C{^1^H} NMR (101 MHz, D_2_O): δ: 81.9 (C-4), 80.1 (C-3), 67.4 (C-5), 65.0 (C-6), 63.4 (C-2), 62.9 (C-1).

MP: 134–136 °C (Lit 139–142.5 °C).^46^

[α]_D_^25.0^ = +32.6 (*c* 1.0 in H_2_O), (Lit. [α]_D_^20.0^ = +27.6 (*c* 1.2 in H_2_O))^42^

HRMS(ESI/Q-TOF) *m*/*z*: [M + H]+ calcd for C_6_H_14_NO_4_ 164.0917; found, 164.0917.

### 3,4-Di-*O*-acetyl-5-bromo-5-deoxy-1,2-*O*-isopropylidene-α-l-tagatopyranose (**29**)

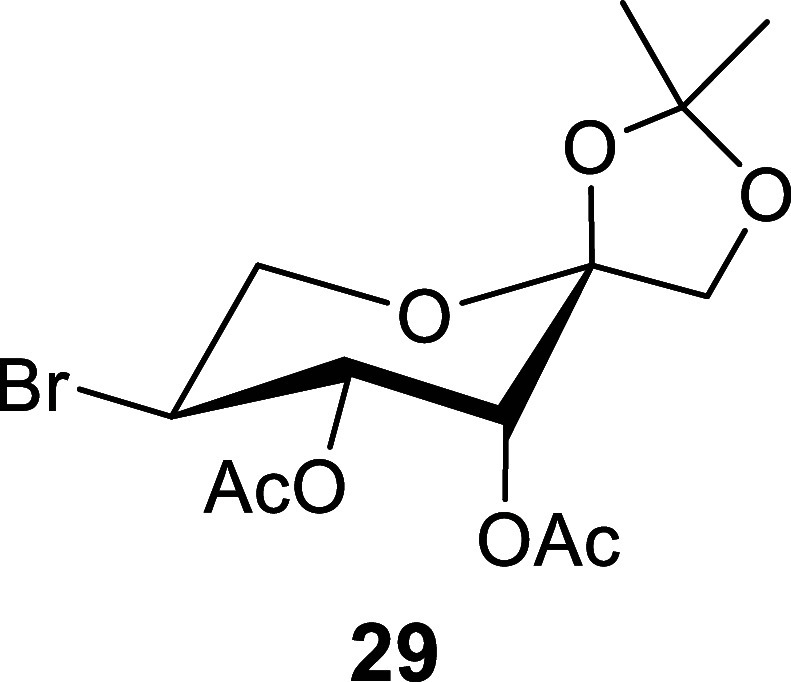



To a round-bottom flask containing 1,2-*O*-isopropylidene-β-d-psicopyranose (**27**)^51^ (1.24 g, 5.65 mmol) was added pyridine (30 mL) and triphenylphosphine (4.45 g, 16.95 mmol, 3 equiv) before the solution was cooled in an ice bath. Once cool, CBr_4_ (5.62 g, 16.95 mmol, 3 equiv) was added in one portion and stirred for 5 min before the reaction vessel was heated to 80 °C for 2 h. After 2 h, the reaction vessel was allowed to cool to rt. before excess MeOH (20 mL) was added slowly and the solution stirred for 15 min. After 15 min, the solution was concentrated in vacuo to afford a crude dark syrup. The syrup was azeotropically dried in vacuo with toluene (3 × 50 mL), before being taken up with excess Ac_2_O in pyridine (15:15 mL v/v), and stirred overnight. The next day, the solution was cooled in an ice bath before excess Ac_2_O was degraded with 10 mL of MeOH. The solution was stirred for 15 min before being concentrated in vacuo to afford a crude dark syrup. The syrup was purified via flash column chromatography [6:1 hexanes/EtOAc] to afford the title compound as a colorless syrup, which crystallized upon extended drying. (1.84 g, 5.03 mmol, 89%)

^1^H NMR (400 MHz, CDCl3): δ 5.31 (dd, *J* = 10.8, 3.2 Hz, H-4, 1H), 5.19 (d, *J* = 3.2 Hz, H-3, 1H), 4.14 (td, *J* = 10.9, 6.0 Hz, H-5, 1H), 4.03 (d, *J* = 11.3 Hz, H-6a, 1H), 4.00–3.94 (m, H-6b, 1H), 3.91 (d, *J* = 9.3 Hz, H-1a 1H), 3.76 (d, *J* = 9.3 Hz, H-1b 1H), 2.10 (s, OAc, CH_3_, 3H), 2.01 (s, OAc, CH_3_, 3H), 1.47–1.44 (s, Acetonide, CH_3_, 3H), 1.38 (s, Acetonide, CH_3_, 3H).

^13^C NMR (101 MHz, CDCl3): δ 169.7 (OAc, C=O), 169.4(OAc, C=O), 113.4 (Acetonide, C), 103.7 (C-2), 73.0 (C-1), 72.1(C-3), 71.9 (C-4), 64.4 (C-6), 43.0 (C-5), 26.7 (Acetonide, CH_3_), 26.2 (Acetonide, CH_3_), 20.7 (OAc, CH_3_), 20.6 (OAc, CH_3_).

MP: 65–69 °C.

[α]_D_^21.4^ = −32.3 (*c* = 1.0 in CH_2_Cl_2_).

HRMS(ESI/Q-TOF) *m*/*z*: [M + NH_4_]+ calcd for C_13_H_23_N^79^BrO_7_ 384.0637; found, 384.0633.

### 3,4-Di-*O*-acetyl-5-bromo-5-deoxy-1,2-*O*-isopropylidene-α-l-psicopyranose (**30**)

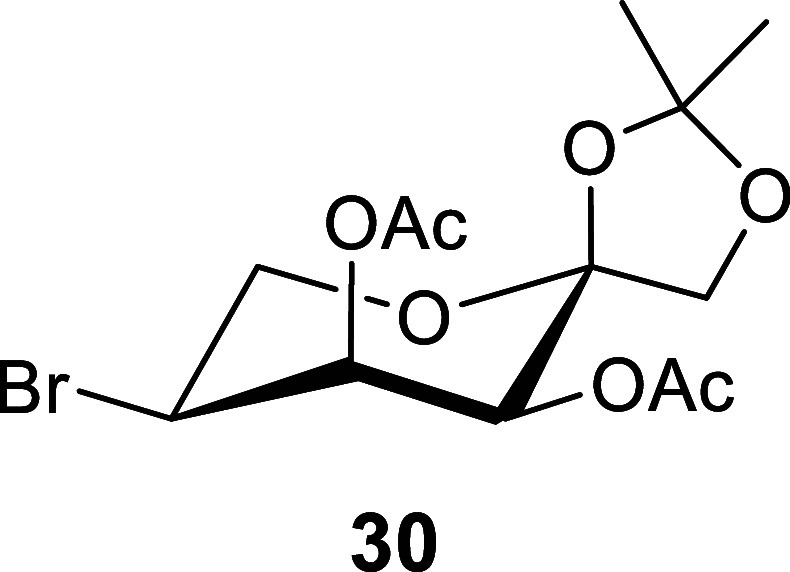



To a round-bottom flask containing 1,2-*O*-isopropylidene-β-d-tagatopyranose (**28**)^50^ (0.10 g, 0.45 mmol) was added triphenylphosphine (0.31 g, 1.35 mmol). The solids were dissolved in pyridine (10 mL), and the reaction vessel was placed in an ice bath. Once cooled, CBr_4_ (0.45 g, 1.35 mmol) was added in a single portion and allowed to stir for 10 min before being heated to 80 °C. After 24 h at reflux, TLC indicated some consumption of the starting material to a single higher Rf spot. The reaction vessel was allowed to cool to rt before an excess of methanol (5 mL) was added. The syrup was azeotropically dried in vacuo with toluene (3 × 50 mL), before being taken up with excess Ac_2_O in pyridine (15:15 mL v/v), and stirred overnight. The next day the solution was cooled in an ice bath before excess Ac_2_O was degraded with 10 mL of MeOH. The solution was stirred for 15 min before being concentrated in vacuo to afford a crude dark syrup. The crude syrup was purified via flash column chromatography [6:1 hexanes/EtOAc] to afford the title compound (**30**) as a brown amorphous solid (61 mg, 0.167 mmol, 37%)

^1^H NMR (400 MHz, CDCl_3_): δ 5.43 (d, *J* = 3.4 Hz, H-3, 1H), 5.27 (dd, *J* = 5.7, 3.4 Hz, H-4, 1H), 4.44 (dd, *J* = 13.0, 3.0 Hz, H-6a, 1H), 4.16–4.07 (m, H-1a, H-5, 2H), 3.94 (d, *J* = 9.2 Hz, H-1b, 1H), 3.71 (ddd, *J* = 13.0, 4.7, 0.9 Hz, H-6b, 1H), 2.10 (s, Acetonide, CH_3_, 3H), 2.09 (s, Acetonide, CH_3_, 3H), 1.50 (s, OAc, CH_3_ 3H), 1.39 (s, OAc, CH_3_, 3H).

^13^C NMR (101 MHz, CDCl_3_): δ 170.1 (OAc, C=O), 169.8 (OAc, C=O), 112.6 (Acetonide, C), 103.5 (C-2), 71.7 (C-1), 70.8(C-4), 66.9 (C-3), 63.7 (C-6), 44.5 (C-5), 26.6 (Acetonide, CH_3_), 26.1 (Acetonide, CH_3_), 20.9 (OAc, CH_3_), 20.8 (OAc, CH_3_).

[α]_D_^21.4^ = −26.6 (*c* 1.0 in CH_2_Cl_2_).

HRMS(ESI/Q-TOF) *m*/*z*: [M + NH_4_]+ calcd for C_13_H_23_N^79^BrO_7_ 384.0658; found, 386.0643; [M + NH_4_]+ calcd for C_13_H_23_N^81^BrO_7_ 386.0637; found, 386.0636.

## Data Availability

The data underlying this study are available in the published article and its Supporting Information.

## Supporting Information Available

^1^H//^13^C/^1^H{^13^C} NMR spectra and mass spectra of compounds (PDF)
