# Supplementary material for: Multigram-Scale Synthesis of 2,5-Dideoxy-2,5-imino-d-mannitol (DMDP) and 2,5-Dideoxy-2,5-imino-d-glucitol (DGDP) from d-Fructose and l-Sorbose Using a Regioselective Appel Reaction
Source: J Org Chem. 2025 Feb 5;90(6):2288–97. doi: 10.1021/acs.joc.4c02667 (PMC11833879; doi:10.1021/acs.joc.4c02667)
Supplement: Supplementary file 1 — jo4c02667_si_001.pdf [file jo4c02667_si_001.pdf]

Supporting Information

Multigram-scale synthesis of 2,5-dideoxy-2,5-imino-D-mannitol (DMDP) and 2,5-dideoxy-2,5-imino-D-glucitol (DGDP) from D-fructose and L-sorbose using a regioselective Appel reaction

Peter Sunde-Brown*,[1,2] Gavin J. Miller,[2] Todd A. Houston,[1,3]

[1] Institute for Glycomics, Griffith University, Gold Coast Campus, 4215, QLD, Australia

[2] School of Chemical and Physical Sciences and Centre for Glycoscience, Keele University, Keele, Staffordshire, ST5 5GH, UK

[3] School of Environment and Science, Griffith University, Gold Coast Campus, 4215, QLD, Australia

p.sunde-brown@keele.ac.uk

Contents

$^{1}H/^{13}C\{1H\}$/HSQCDEPT NMR spectra and Mass spectra of Compounds:..S-2

Compound 11: 2-Chloroethyl 5-bromo-5-deoxy-α-L-sorbopyranoside..S-2

Compound 13: 2-Chloroethyl 1,3,4-tri-*O*-acetyl-5-bromo-5-deoxy-α-L-sorbopyraniside………………S-4

Compound 14: 2-Azidoethyl 1,3,4-tri-*O*-acetyl-5-azido-5-deoxy-β-D-fructopyranoside……………...S-6

Compound 12: 5-Azido-5-deoxy-α,β-D-fructopyranose…………………………………………………………….S-8

Compound 4: 2,5-Dideoxy-2,5-imino-D-mannitol (DMDP)……………………………………………………...S-110

Compound 21: Methyl 1,3,4-tri-*O*-acetyl-5-bromo-5-deoxy-β-D-fructopyranoside……………………S-12

Compound 22: Methyl 1,3,4-tri-*O*-acetyl-5-azido-5-deoxy-α-L-sorbopyranoside……………………….S-14

Compound 23: 5-azido-5-deoxy-α-L-sorbopyranose…………………………………………………………………S-16

Compound 18: 2,5-Dideoxy-2,5-imino-D-glucitol……………………………………………………………………….S-18

Compound 29: 3,4-Di-*O*-acetyl-5-bromo-5-deoxy-1,2-*O*-isopropylidene-α-L-tagatopyranose……S-20

Compound 30: 3,4-Di-*O*-acetyl-5-bromo-5-deoxy-1,2-*O*-isopropylidene-α-L-psicopyranose………S-23

Cautionary Note: The following manuscript outlines a method which forms 2-azidoethanol as a reaction by-product after acidic hydrolysis which is a potentially explosive material. Care should be taken when handling this material. The organic phase should not be concentrated and the solution should instead be treated with excess triphenylphosphine and allowed to stir for >24 h to decompose the organic azide into the safe to handle iminophosphorane which can be disposed of into halogenated waste

$^{1}H/^{13}C\{1H\}$ NMR spectra and Mass spectra of Compounds:

Compound 11: 2-Chloroethyl 5-bromo-5-deoxy-α-L-sorbopyranoside

^{1}H NMR (400 MHz, MeOD)

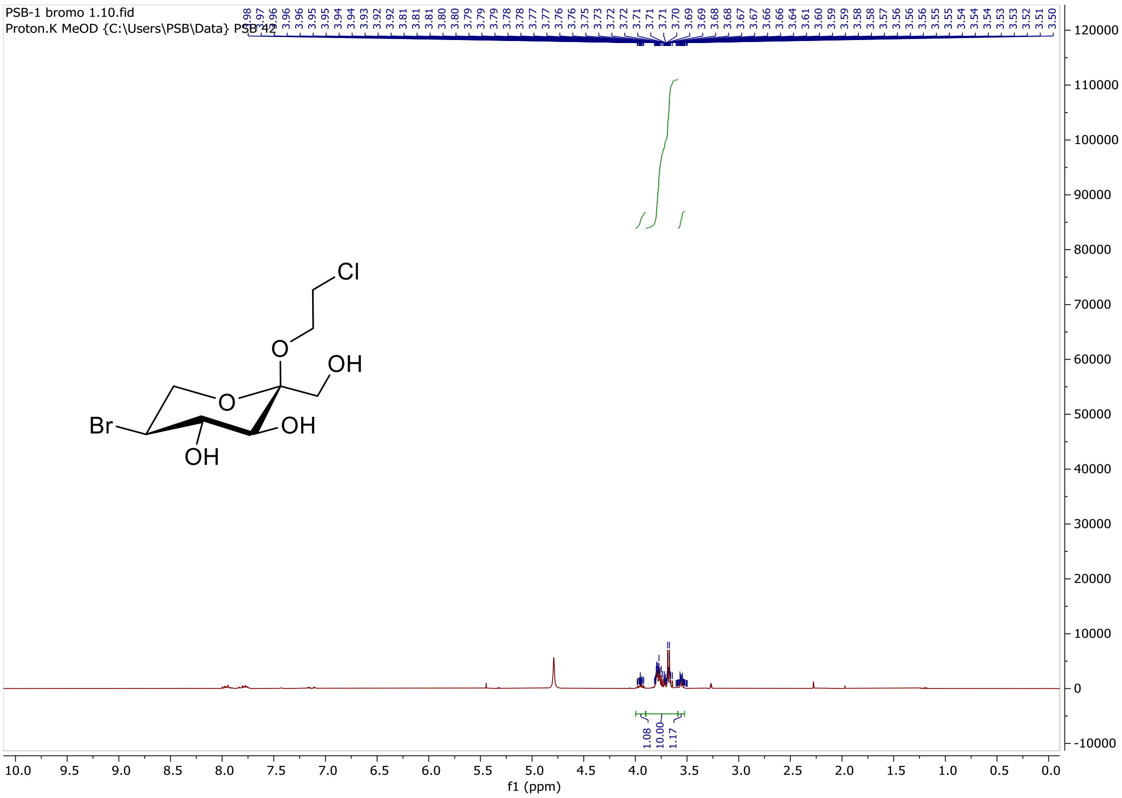

$^{13}C\{1H\}$ NMR (100 MHz, MeOD)

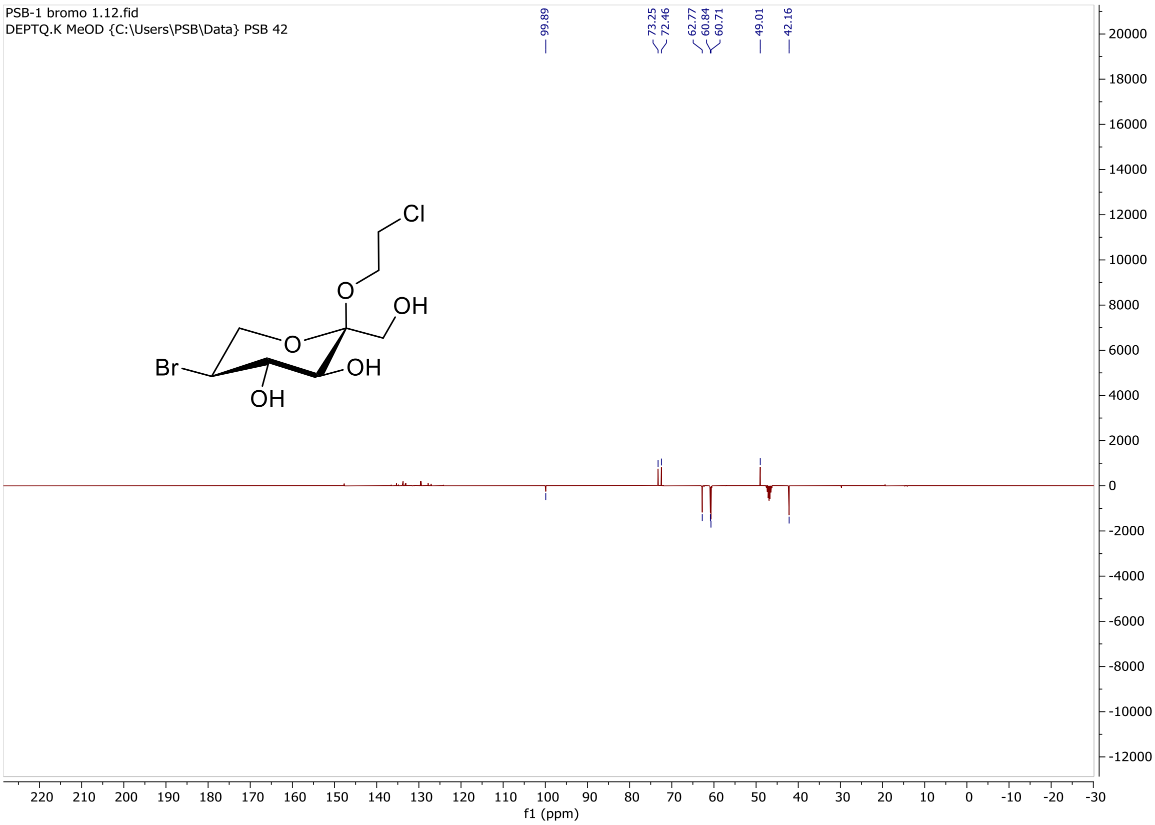

^{1}HSQC{^{13}CDEPTQ} NMR (400 MHz, MeOD)

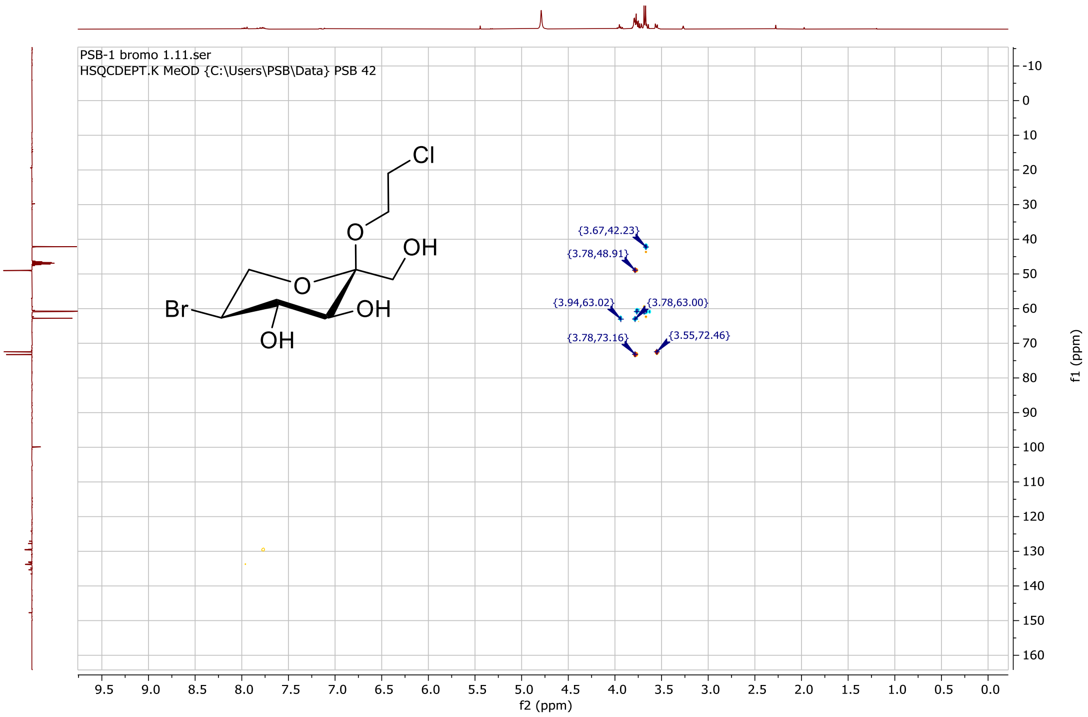

HRMS

Mass Spectrum SmartFormula Report

Analysis Info

Analysis Name	D:\Data\Wendy\20230329\sample 2_000001.d	Acquisition Date	3/29/2023 11:12:33 AM
Method	lmw_DI_20211027	Operator	Admin
Sample Name	sample 2	Instrument	solariX XR
Comment			

Acquisition Parameter

Acquisition Mode	Single MS	Acquired Scans	8	Calibration Date	Wed Mar 29 09:51:25
		No. of Cell Fills	1	Data Acquisition Size	2097152
Broadband Low Mass	98.3 m/z			Data Processing Size (SI)	4194304
Broadband High Mass	2000.0 m/z			Apodization	Full-Sine
Source Accumulation	0.000 sec				
Ion Accumulation Time	0.200 sec				

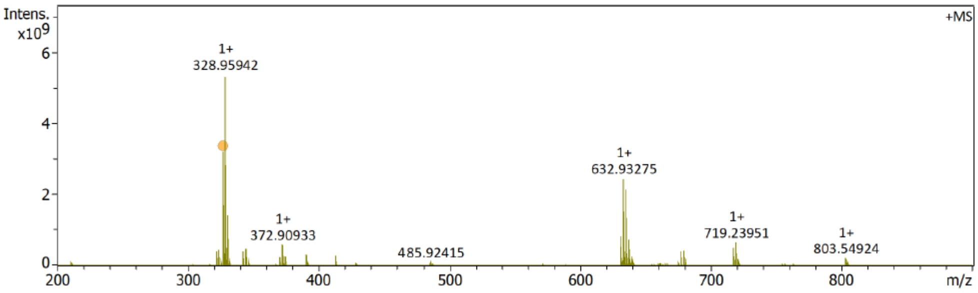

Meas. m/z	#	Ion Formula	Score	m/z	err [ppm]	Mean err [ppm]	mSigma	rdb	e⁻ Conf	N-Rule
326.961525	1	C8H14BrClNaO5	72.57	326.960535	-3.0	-3.0	62.8	6.0	even	ok

Compound 13: 2-Chloroethyl 1,3,4-tri-*O*-acetyl-5-bromo-5-deoxy-α-L-sorbopyranoside

^{1}H NMR (400 MHz, $CDCl_3$)

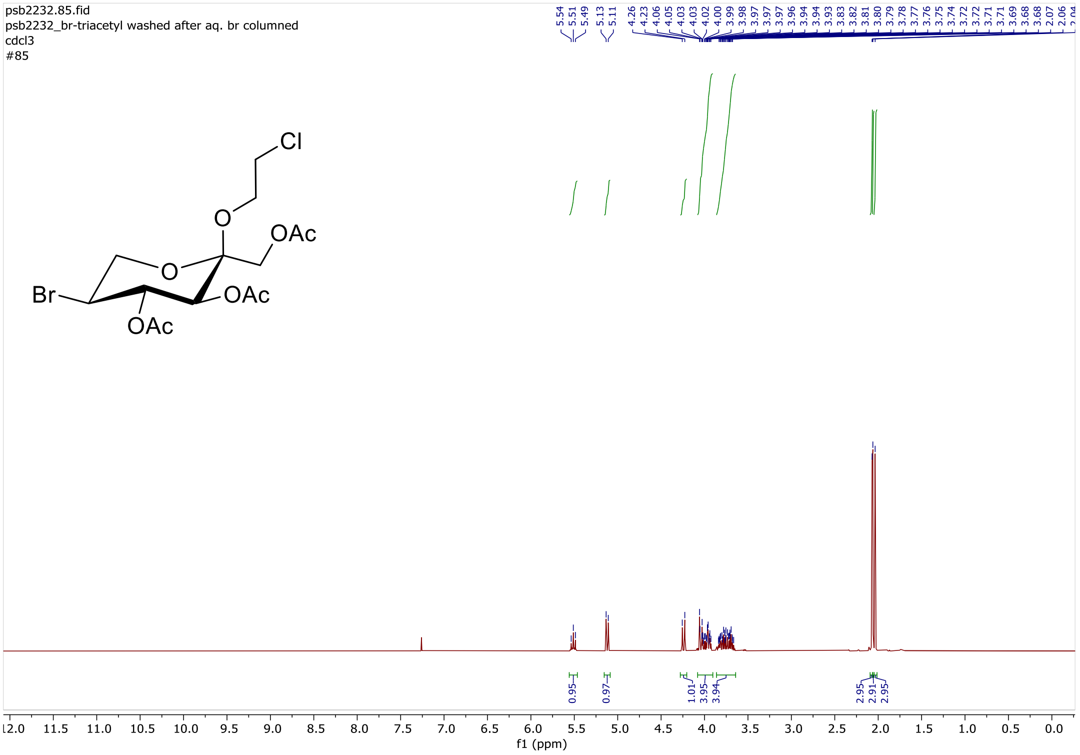

^{13}C{1H} NMR (100 MHz, $CDCl_3$)

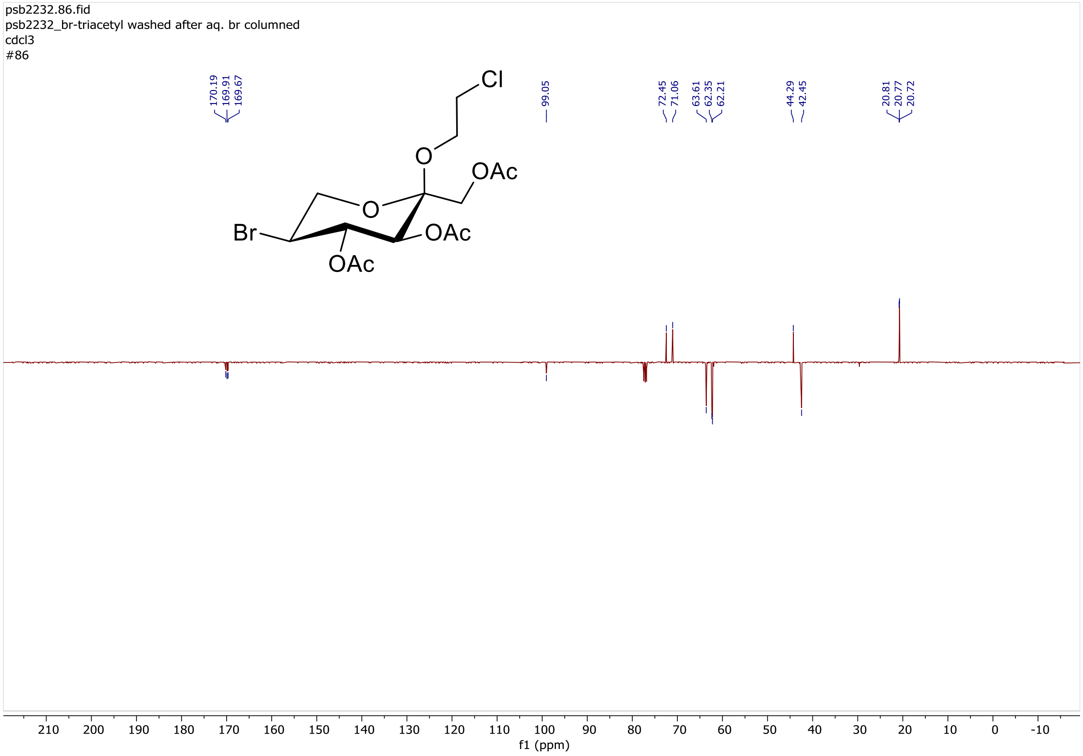

HRMS

Mass Spectrum SmartFormula Report

Analysis Info

Analysis Name	D:\Data\Wendy\20230414\sample 3_000001.d	Acquisition Date	4/14/2023 9:19:46 AM
Method	lmw_DI_20211027	Operator	Admin
Sample Name	sample 3	Instrument	solariX XR
Comment			

Acquisition Parameter

Acquisition Mode	Single MS	Acquired Scans	8	Calibration Date	Fri Apr 14 09:16:58 2023
		No. of Cell Fills	1	Data Acquisition Size	2097152
Broadband Low Mass	98.3 m/z			Data Processing Size (SI)	4194304
Broadband High Mass	2000.0 m/z			Apodization	Full-Sine
Source Accumulation	0.000 sec				
Ion Accumulation Time	0.300 sec				

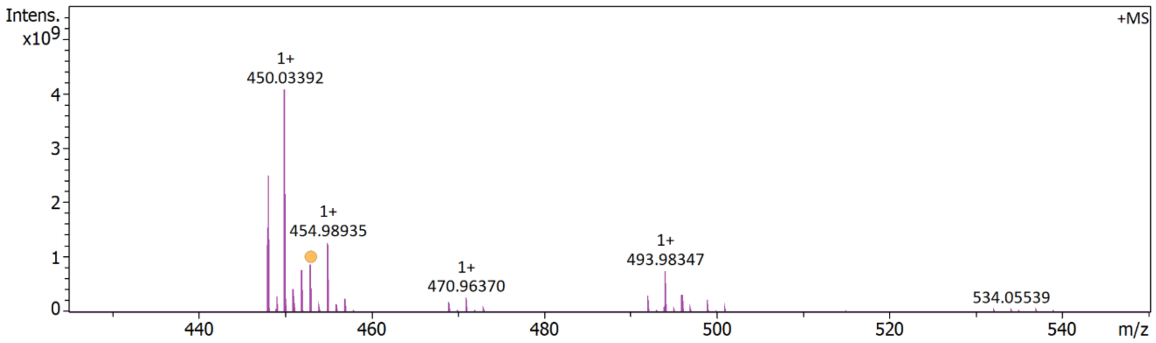

Meas. m/z	#	Ion Formula	Score	m/z	err [ppm]	Mean err [ppm]	mSigma	rdb	e⁻ Conf	N-Rule
452.991831	1	C14H20BrClNaO8	100.00	452.992229	0.9	0.6	42.2	9.0	even	ok

Compound 14: 2-Azidoethyl 1,3,4-tri-*O*-acetyl-5-azido-5-deoxy-β-D-fructopyranose

^{1}H NMR (400 MHz, $CDCl_3$)

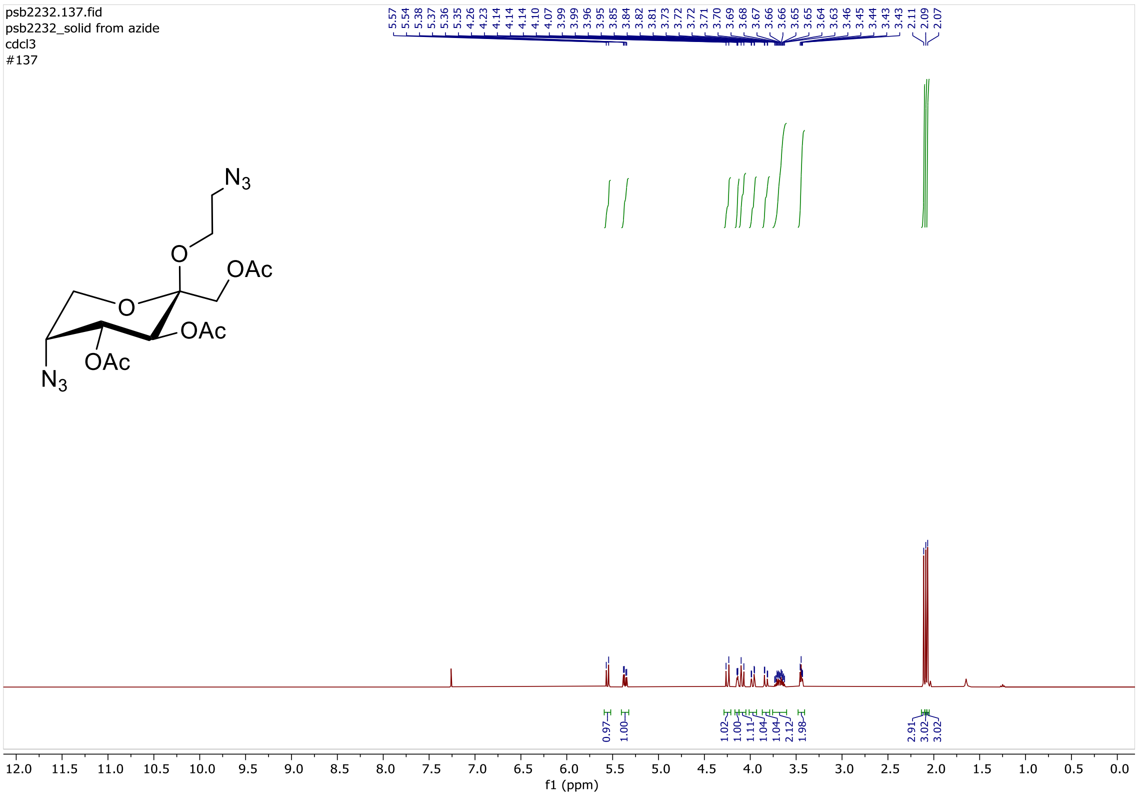

^{13}C{1H} NMR (100 MHz, $CDCl_3$)

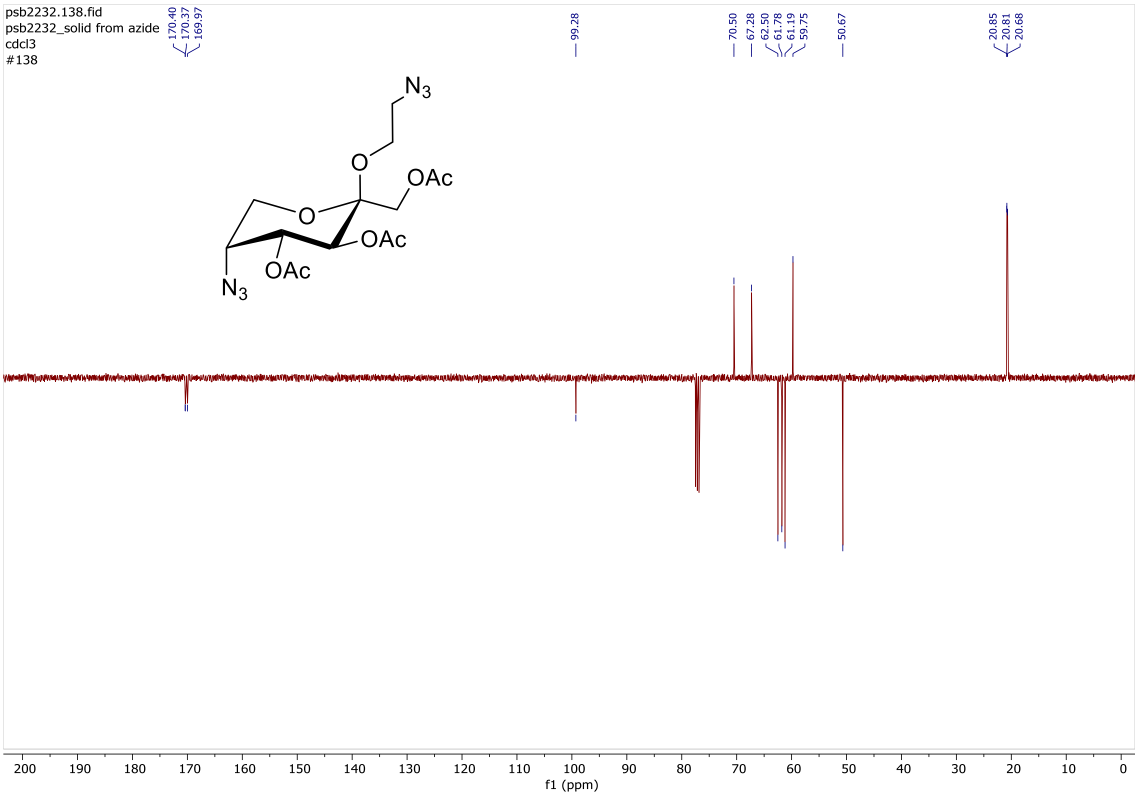

HRMS

Mass Spectrum SmartFormula Report

Analysis Info

Analysis Name	D:\Data\Wendy\20230329\sample 4_000001.d	Acquisition Date	3/29/2023 11:32:40 AM
Method	lmw_DI_20211027	Operator	Admin
Sample Name	sample 4	Instrument	solariX XR
Comment			

Acquisition Parameter

Acquisition Mode	Single MS	Acquired Scans	8	Calibration Date	Wed Mar 29 09:51:25 2023
		No. of Cell Fills	1	Data Acquisition Size	2097152
Broadband Low Mass	98.3 m/z			Data Processing Size (SI)	4194304
Broadband High Mass	2000.0 m/z			Apodization	Full-Sine
Source Accumulation	0.000 sec				
Ion Accumulation Time	0.200 sec				

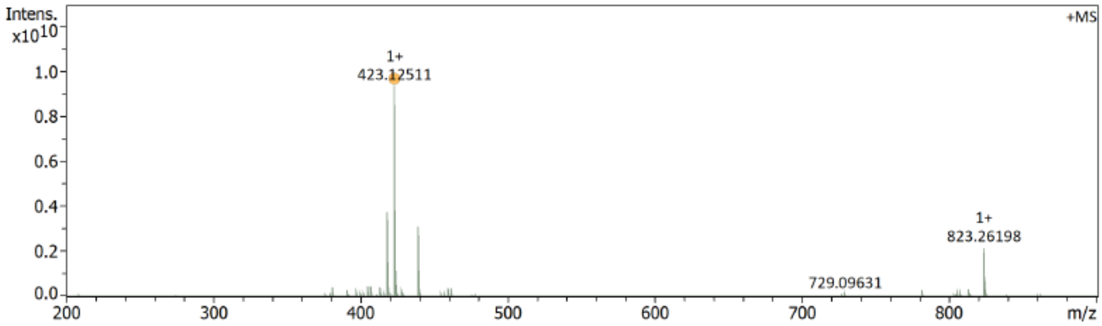

Meas. m/z	#	Ion Formula	Score	m/z	err [ppm]	Mean err [ppm]	mSigma	rdb	e⁻ Conf	N-Rule
423.125108	1	C14H20N6NaO8	100.00	423.123482	-3.8	-4.5	12.9	8.0	even	ok

Compound 12: 5-Azido-5-deoxy-α,β-D-fructopyranose

^{1}H NMR (400 MHz, D_2O)

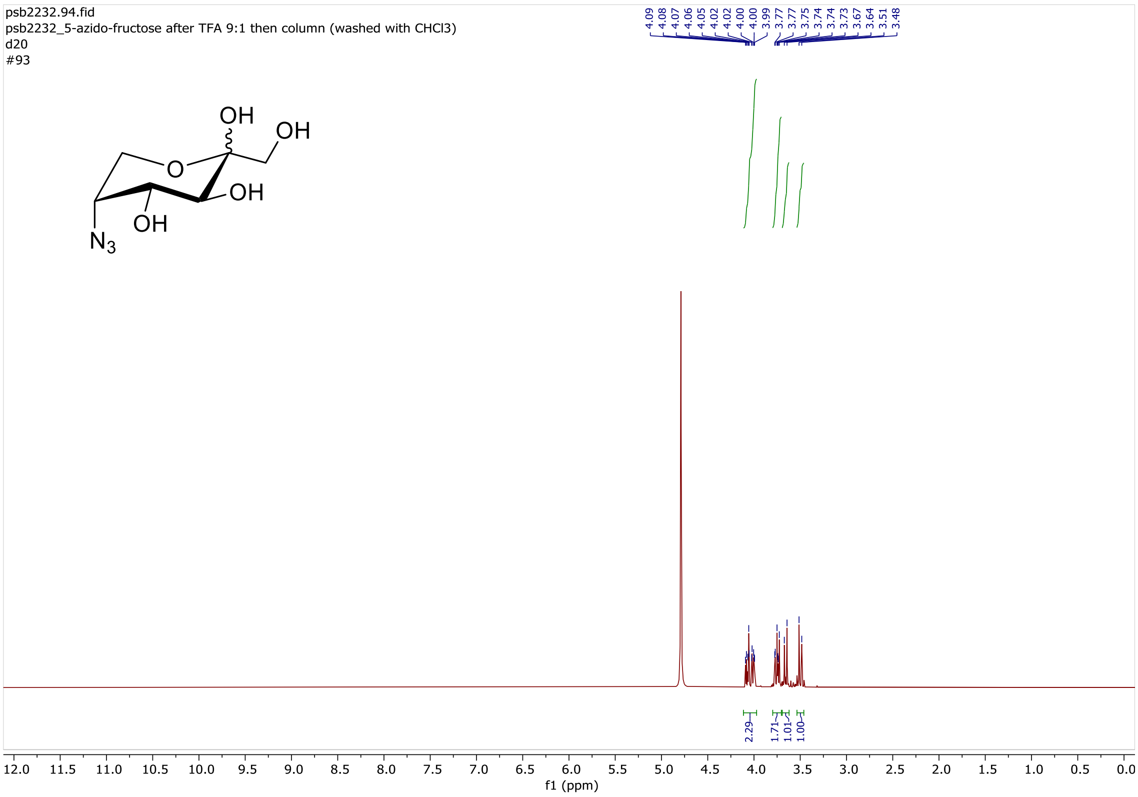

^{13}C{1H} NMR (100 MHz, D_2O)

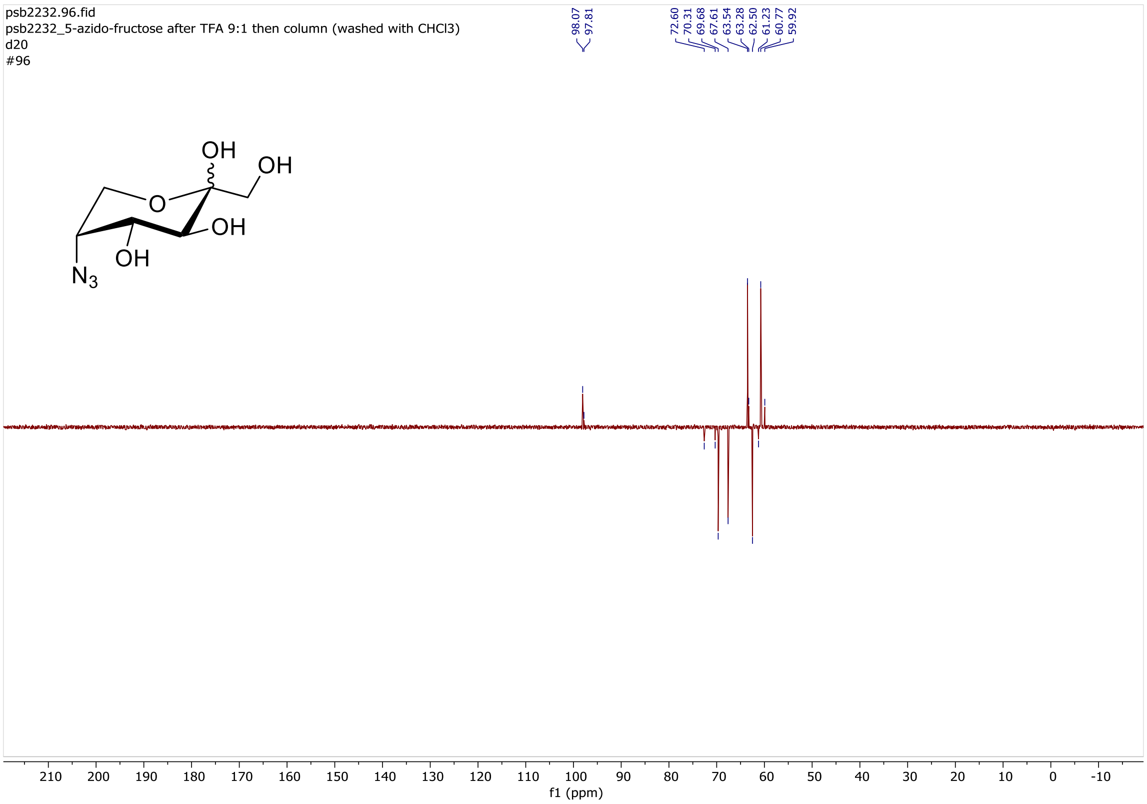

HRMS

Mass Spectrum SmartFormula Report

Analysis Info

Analysis Name	D:\Data\Wendy\20230329\sample 5_000001.d	Acquisition Date	3/29/2023 11:37:40 AM
Method	lmw_DI_20211027	Operator	Admin
Sample Name	sample 5	Instrument	solariX XR
Comment			

Acquisition Parameter

Acquisition Mode	Single MS	Acquired Scans	8	Calibration Date	Wed Mar 29 09:51:25 2023
		No. of Cell Fills	1	Data Acquisition Size	2097152
Broadband Low Mass	98.3 m/z			Data Processing Size (SI)	4194304
Broadband High Mass	2000.0 m/z			Apodization	Full-Sine
Source Accumulation	0.000 sec				
Ion Accumulation Time	0.200 sec				

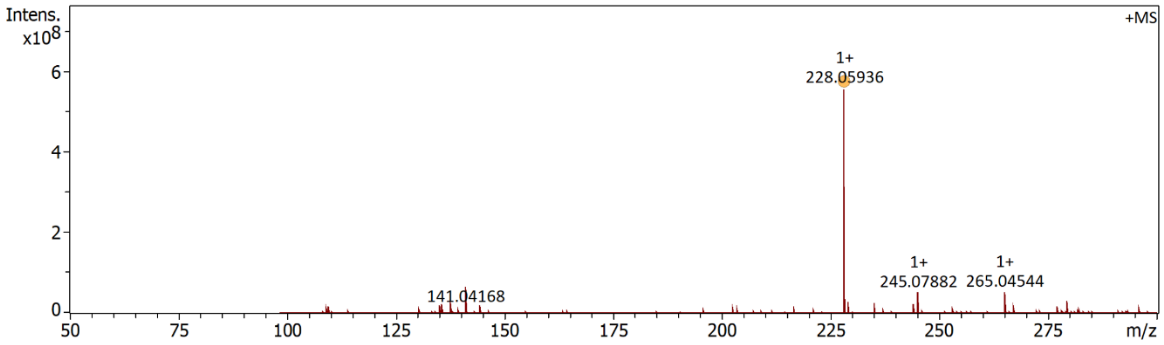

Meas. m/z	#	Ion Formula	Score	m/z	err [ppm]	Mean err [ppm]	mSigma	rdb	e⁻ Conf	N-Rule
228.059358	1	C6H11N3NaO5	100.00	228.059091	-1.2	-1.4	7.0	3.0	even	ok

Compound 4: 2,5-Dideoxy-2,5-imino-D-mannitol (DMDP)

^{1}H NMR (400 MHz, D_2O)

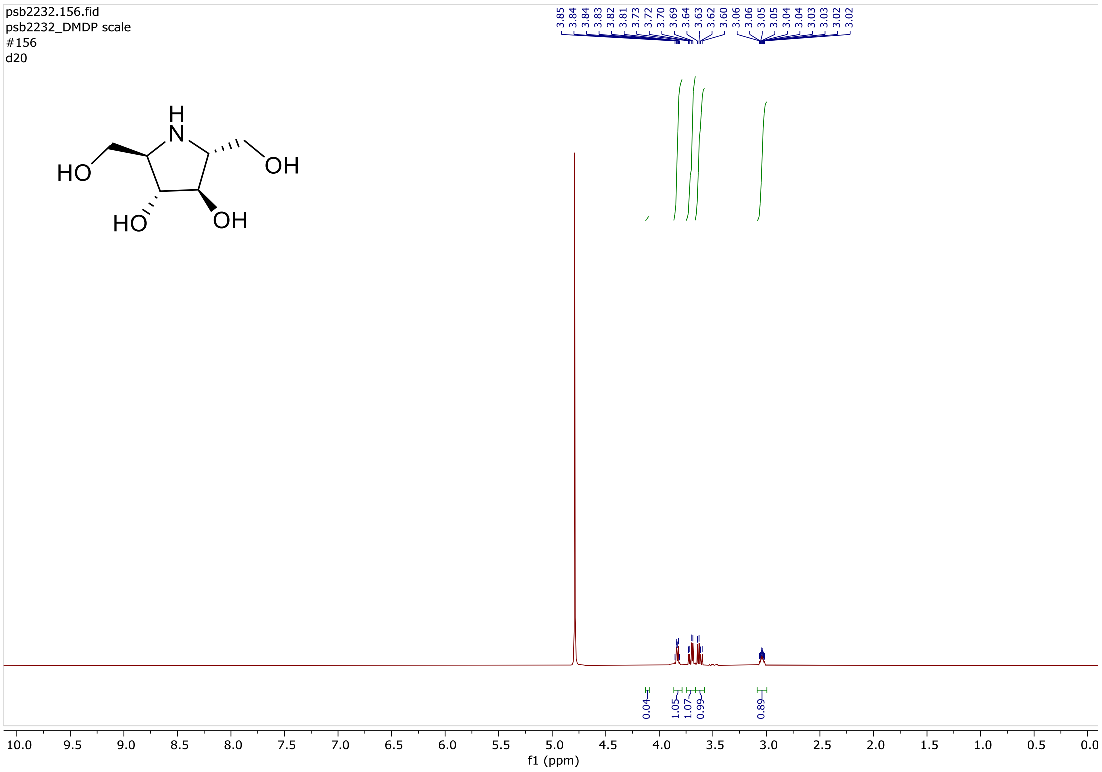

$^{13}C\{1H\}$ NMR (100 MHz, D_2O)

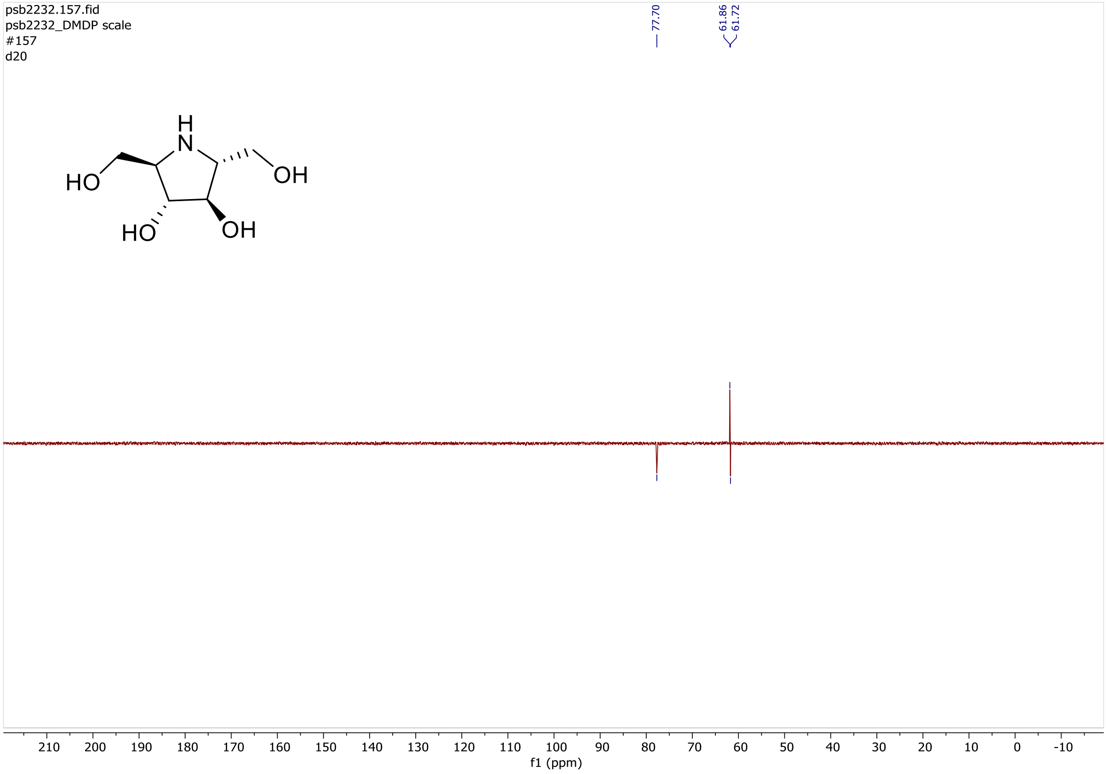

HRMS

Mass Spectrum SmartFormula Report

Analysis Info

Analysis Name	D:\Data\Wendy\20230329\sample 6_000001.d	Acquisition Date	3/29/2023 11:43:39 AM
Method	lmw_DI_20211027	Operator	Admin
Sample Name	sample 6	Instrument	solariX XR
Comment			

Acquisition Parameter

Acquisition Mode	Single MS	Acquired Scans	8	Calibration Date	Wed Mar 29 09:51:25 2023
		No. of Cell Fills	1	Data Acquisition Size	2097152
Broadband Low Mass	98.3 m/z			Data Processing Size (SI)	4194304
Broadband High Mass	2000.0 m/z			Apodization	Full-Sine
Source Accumulation	0.000 sec				
Ion Accumulation Time	0.200 sec				

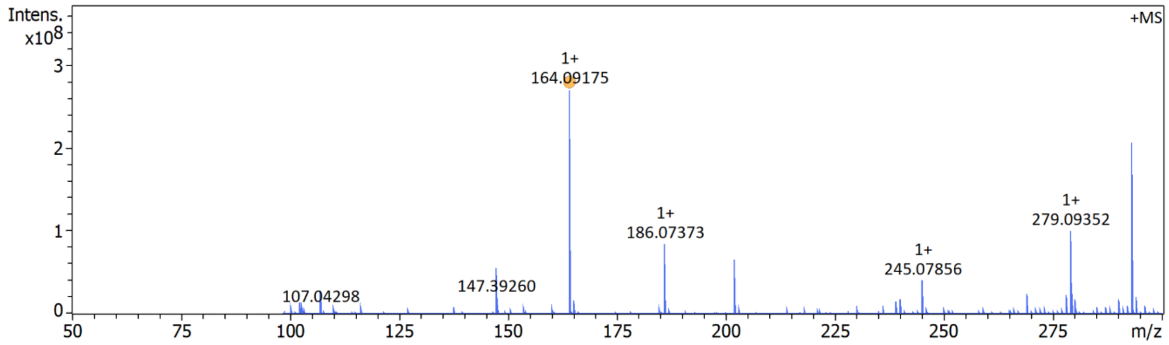

Meas. m/z	#	Ion Formula	Score	m/z	err [ppm]	Mean err [ppm]	mSigma	rdb	e⁻ Conf	N-Rule
164.091752	1	C6H14NO4	100.00	164.091734	-0.1	-0.2	2.0	1.0	even	ok
	2	C7H10N5	43.13	164.093072	8.0	8.0	11.7	6.0	even	ok

Compound 21: Methyl 1,3,4-tri-*O*-acetyl-5-bromo-5-deoxy-β-D-fructopyranoside

^{1}H NMR (400 MHz, $CDCl_3$)

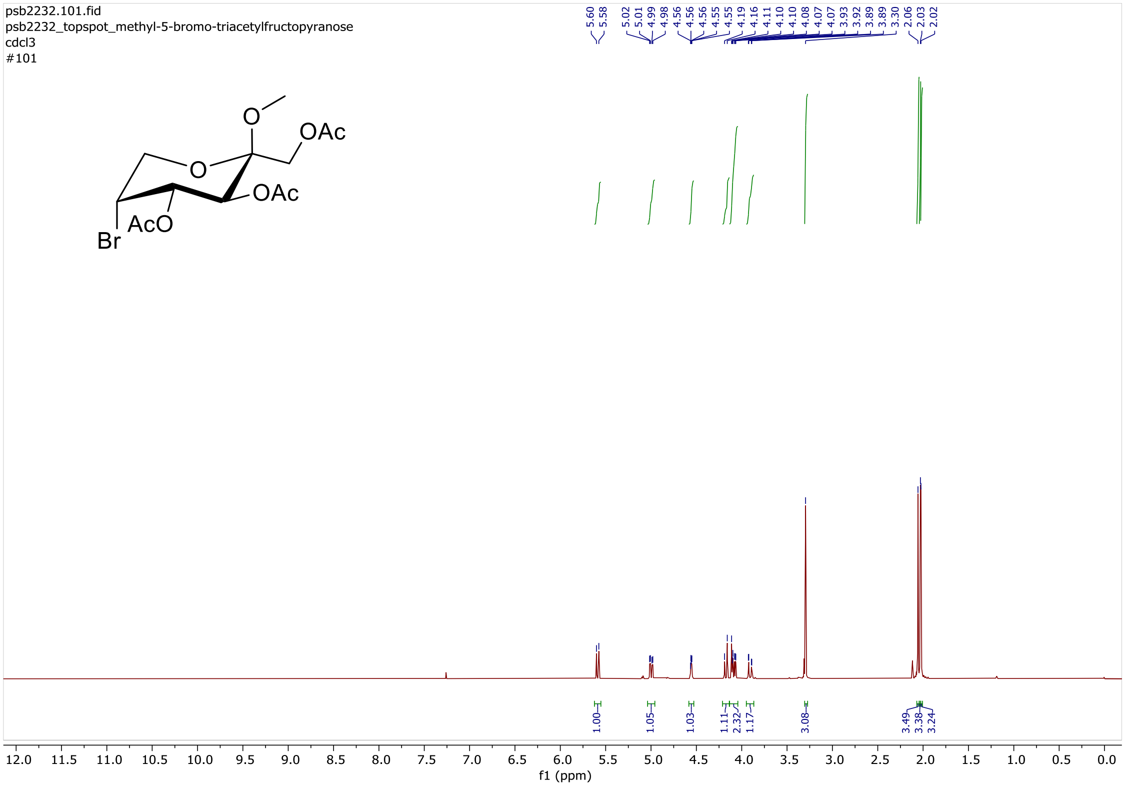

^{13}C{1H} NMR (100 MHz, $CDCl_3$)

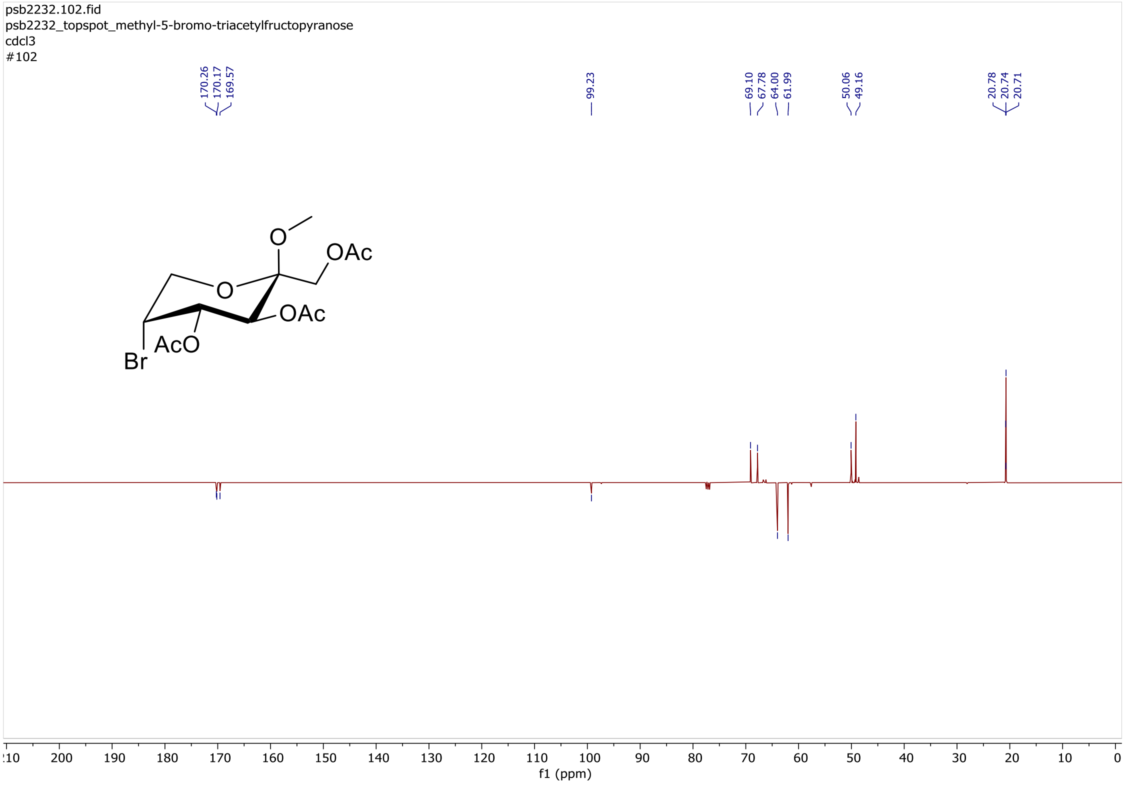

LRMS

ESI-MS Sample Information

Sample ID	: PSB2232_Methyl 5-bromo-triacety	Date Acquire	:3/3/2023 1:59:14 PM
Data File	:PSB2232_Methyl 5-bromo-triacetyl-b-D-fructopyranose pos 330 - 4.lcd	Date Processed	
Injection Volume	: 0.2	Vial	:10
Method File	: 10p.lcm		
Comments	: Peter:PSB2232_Methyl 5-bromo-triacetyl-b-D-fructopyranose<br>(filtered thru 0.22 μm RC filter)<br>dissolved in MeOH (made up to 0.5 mg/mL)<br>20 μL transferred to 980 μL MeOH. (10 ng/mL)<br>MP: 70%AqMeOH+0.1%Formic acid.<br>Flowrate: 0.4 mL/min; 39ºC; 0.2 μL; 770 psi<br>ET: 0.1; DV:0; MS: pos 330 - 450<br>383.19?		

Spectrum

MS Spectrum

POLARITY: Positive

Peak#:1 R.Time:0.193(Scan#:117)
MassPeaks:88
Spectrum Mode:Averaged 0.150-0.250(91-151)
BG Mode:Averaged 0.500-0.700(301-421) Segment 1 - Event 1

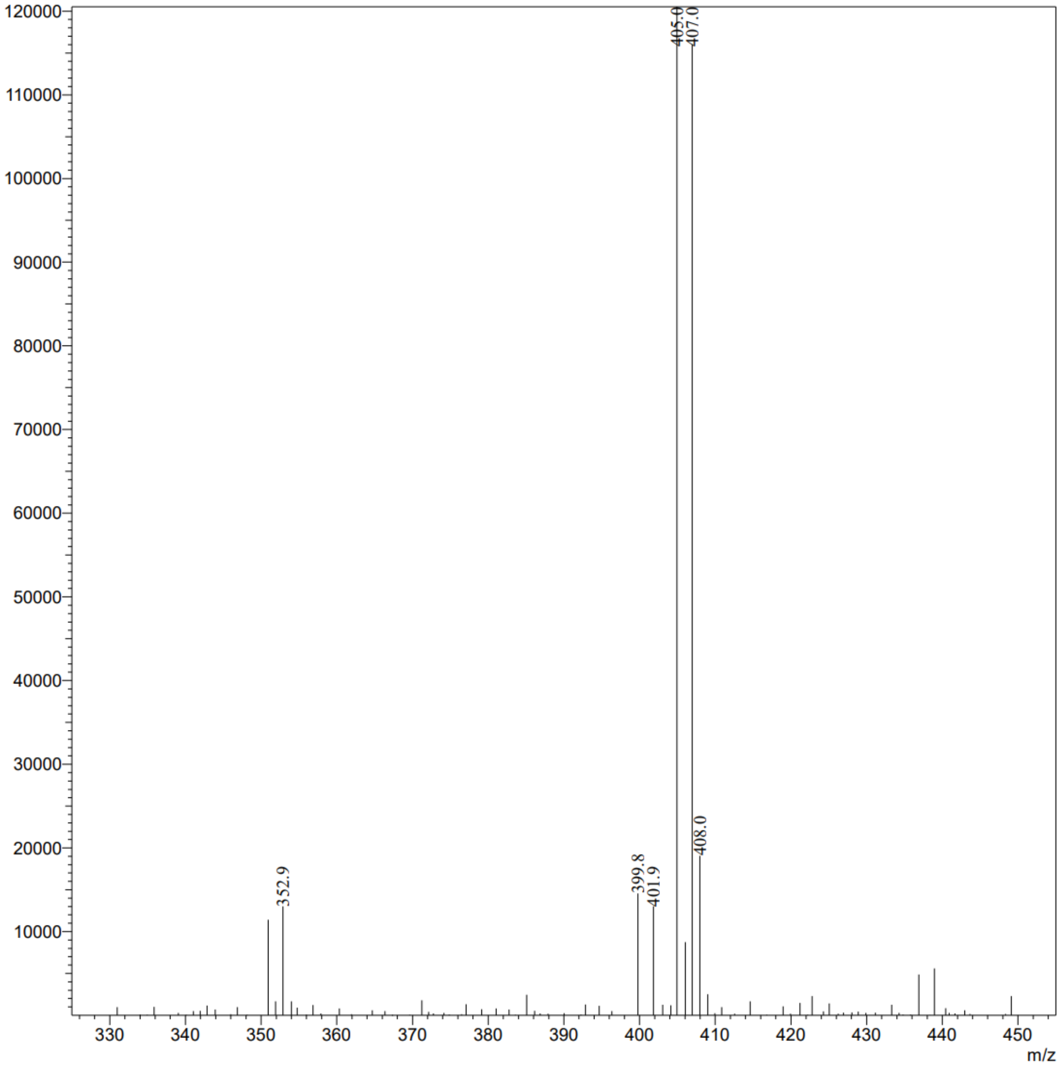

Compound 22: Methyl 1,3,4-tri-O-acetyl-5-azido-5-deoxy-α-L-sorbopyranose

^{1}H NMR (400 MHz, $CDCl_3$)

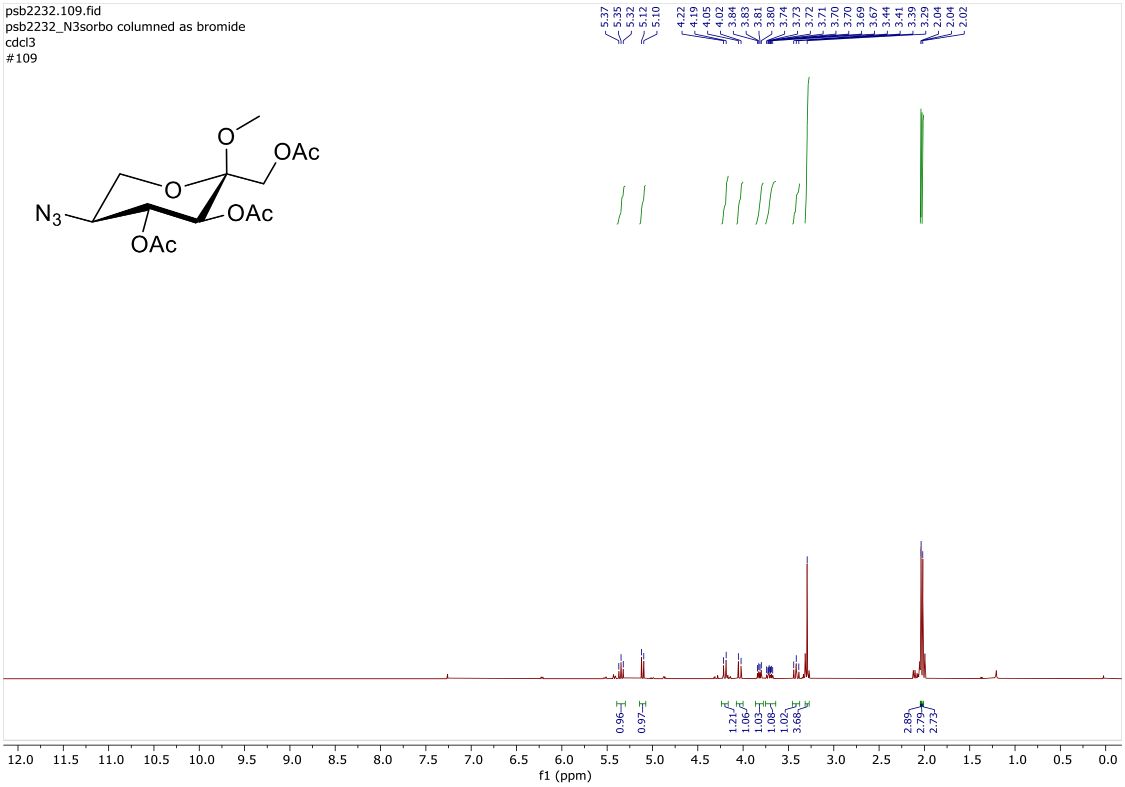

^{13}C{1H} NMR (100 MHz, $CDCl_3$)

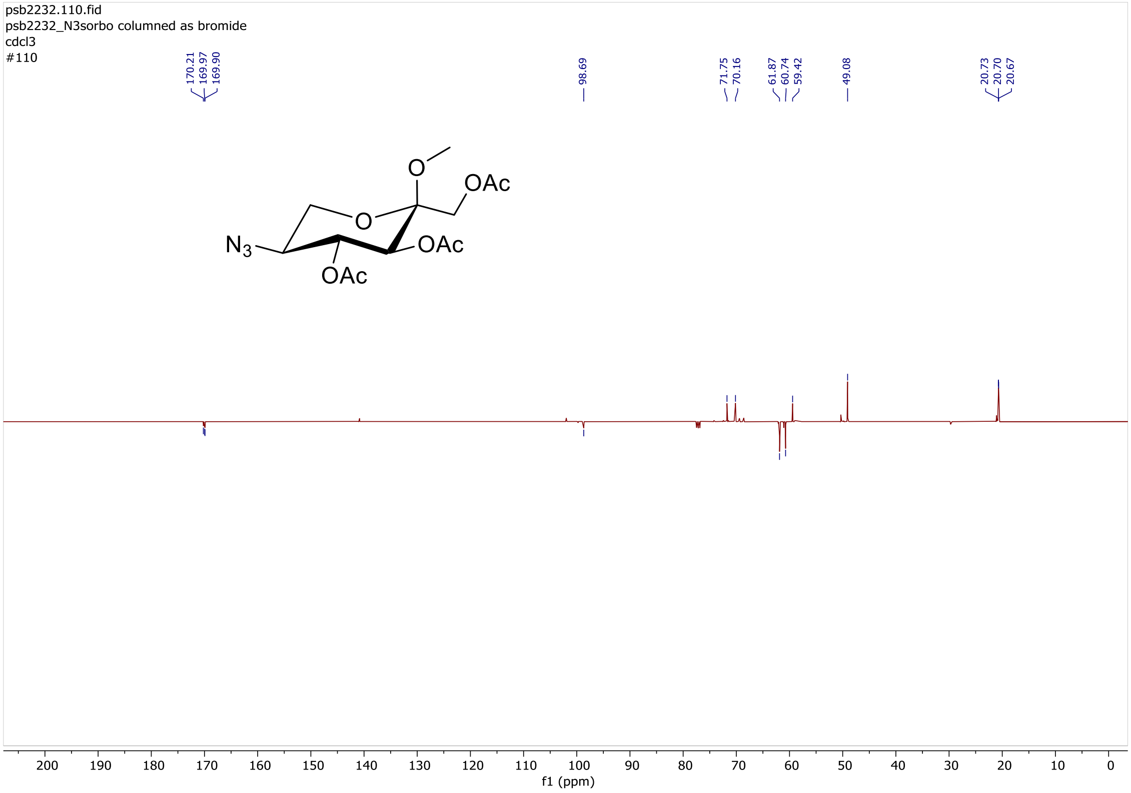

HRMS

Mass Spectrum SmartFormula Report

Analysis Info

Analysis Name	D:\Data\Wendy\20230404\sample 8_000001.d	Acquisition Date	4/4/2023 10:41:02 AM
Method	lmw_low_DI_20211027	Operator	Admin
Sample Name	sample 8	Instrument	solariX XR
Comment			

Acquisition Parameter

Acquisition Mode	Single MS	Acquired Scans	8	Calibration Date	Tue Apr 4 10:23:39 2023
		No. of Cell Fills	1	Data Acquisition Size	1048576
Broadband Low Mass	98.3 m/z			Data Processing Size (SI)	2097152
Broadband High Mass	2000.0 m/z			Apodization	Full-Sine
Source Accumulation	0.000 sec				
Ion Accumulation Time	0.300 sec				

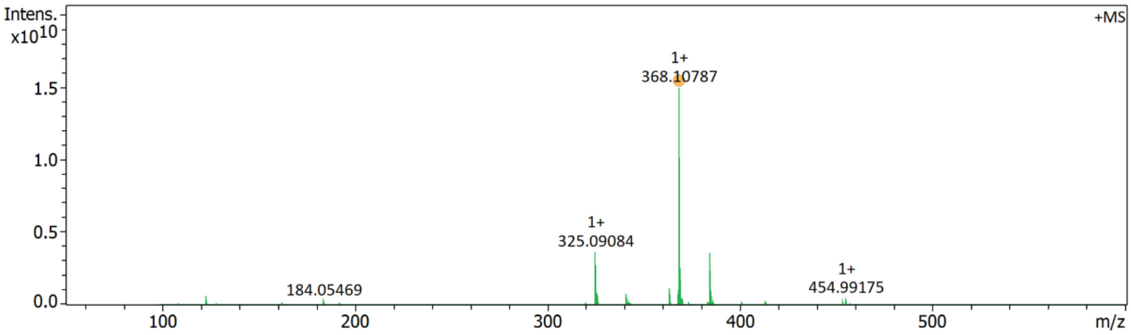

Meas. m/z	#	Ion Formula	Score	m/z	err [ppm]	Mean err [ppm]	mSigma	rdb	e⁻ Conf	N-Rule
368.107866	1	C13H19N3NaO8	100.00	368.106435	-3.9	-3.3	13.5	6.0	even	ok

Compound 23: 5-Azido-5-deoxy-α-L-sorbopyranose

^{1}H NMR (400 MHz, D_2O)

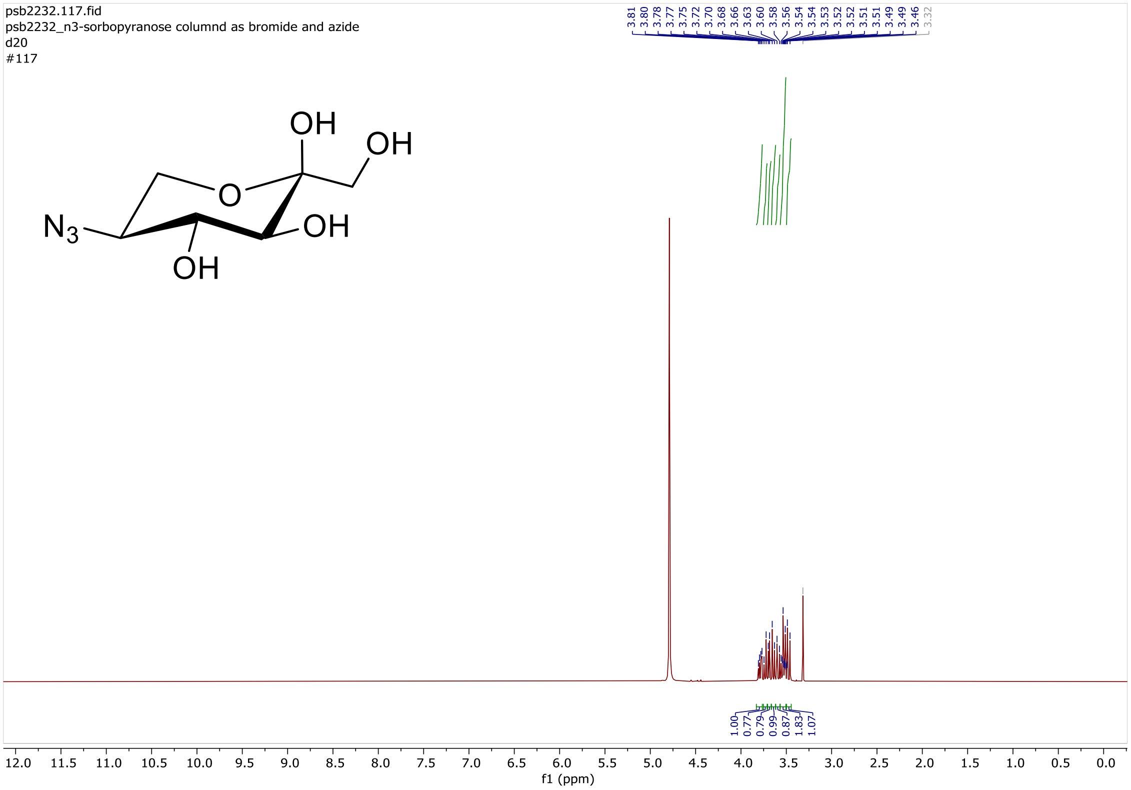

$^{13}C\{^1H\}$ NMR (100 MHz, D_2O)

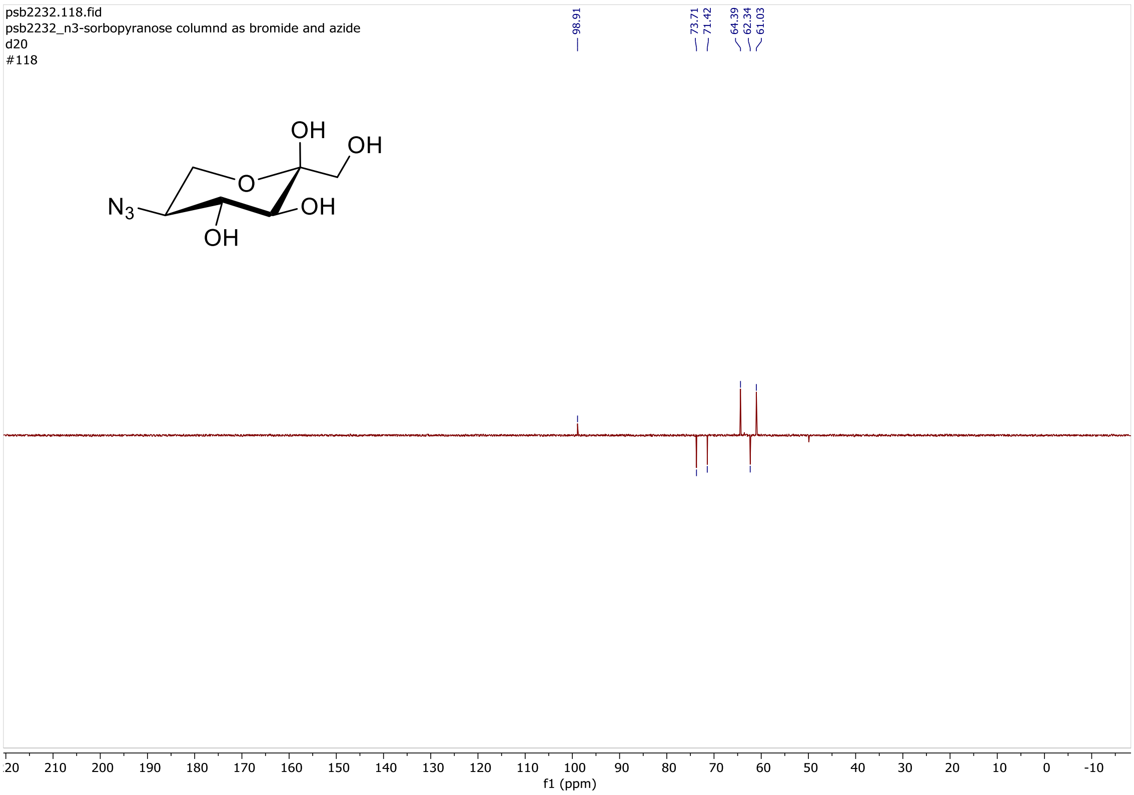

HRMS

Mass Spectrum SmartFormula Report

Analysis Info

Analysis Name	D:\Data\Wendy\20230404\sample 9_000001.d	Acquisition Date	4/4/2023 11:02:14 AM
Method	lmw_low_DI_20211027	Operator	Admin
Sample Name	sample 9	Instrument	solariX XR
Comment			

Acquisition Parameter

Acquisition Mode	Single MS	Acquired Scans	8	Calibration Date	Tue Apr 4 10:23:39 2023
		No. of Cell Fills	1	Data Acquisition Size	1048576
Broadband Low Mass	98.3 m/z			Data Processing Size (SI)	2097152
Broadband High Mass	2000.0 m/z			Apodization	Full-Sine
Source Accumulation	0.000 sec				
Ion Accumulation Time	0.300 sec				

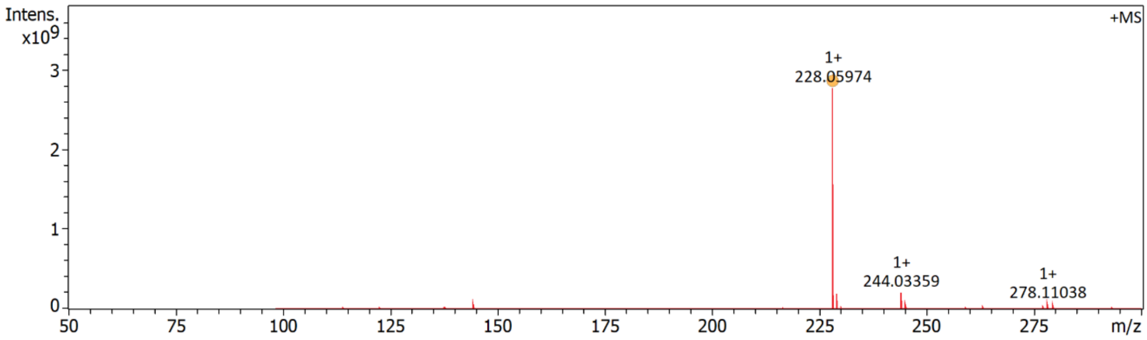

Meas. m/z	#	Ion Formula	Score	m/z	err [ppm]	Mean err [ppm]	mSigma	rdb	e⁻ Conf	N-Rule
228.059743	1	C6H11N3NaO5	100.00	228.059091	-2.9	-2.8	1.9	3.0	even	ok

Compound 18: 2,5-dideoxy-2,5-imino-D-glucitol (DGDP)

^{1}H NMR (400 MHz, D_2O)

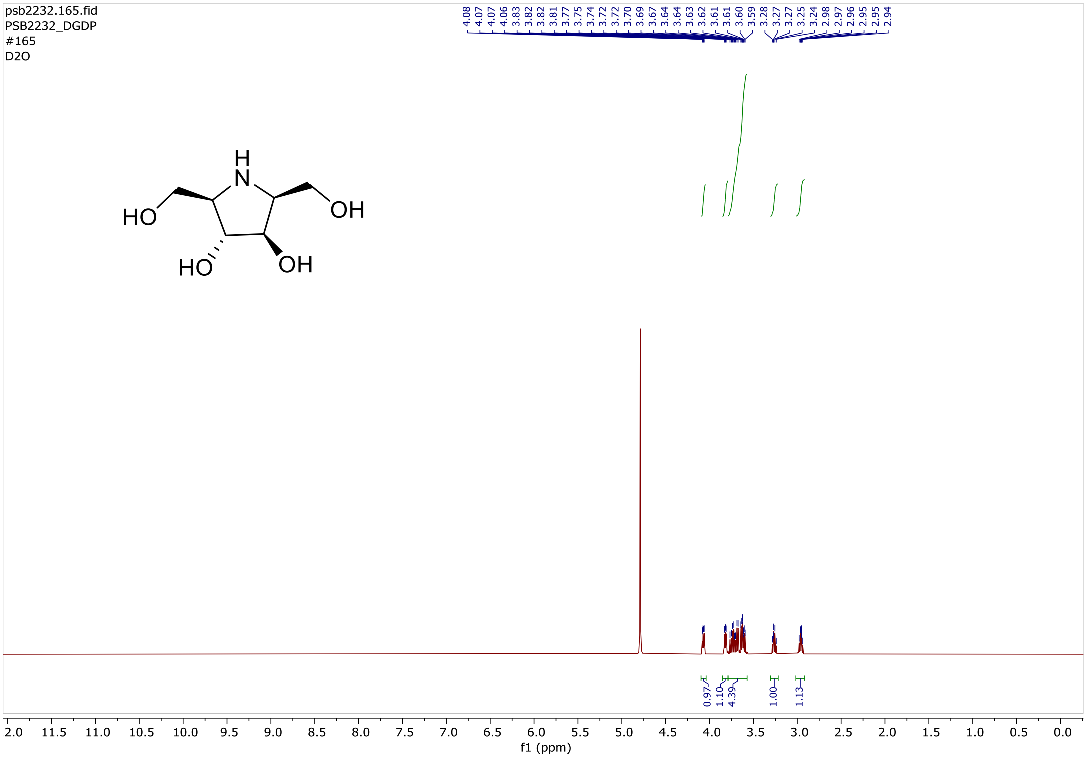

$^{13}C\{^{1}H\}$ NMR (100 MHz, D_2O)

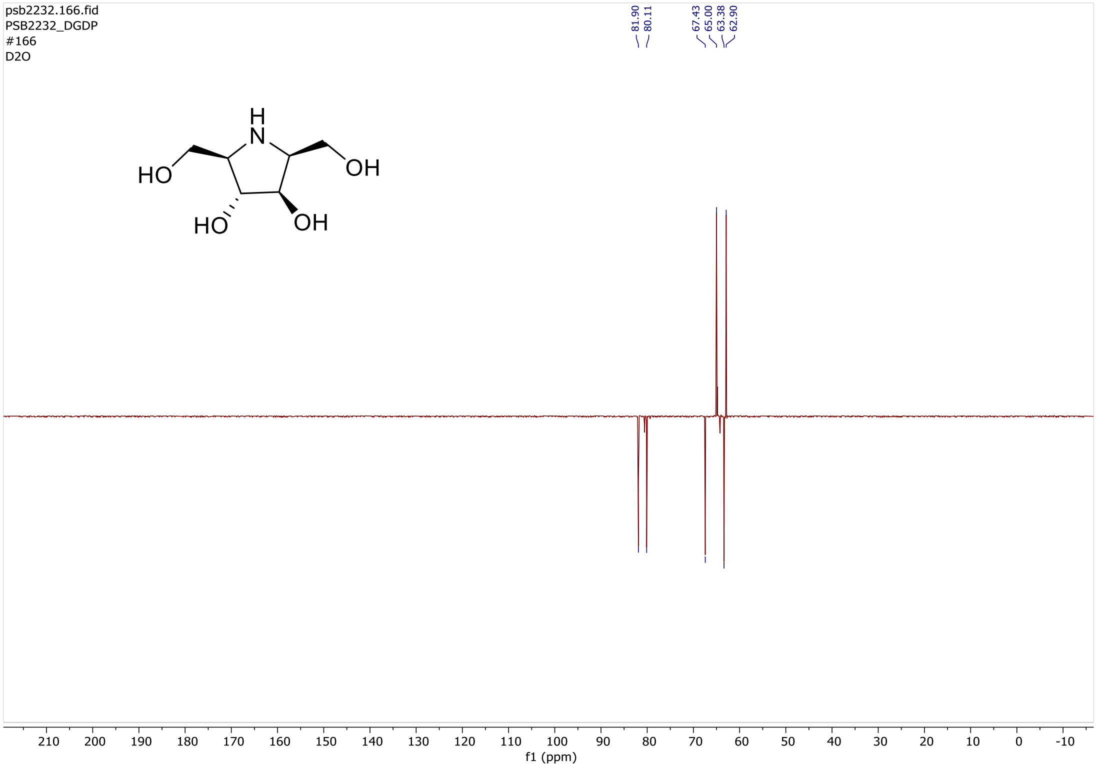

HRMS

Mass Spectrum SmartFormula Report

Analysis Info

Analysis Name	D:\Data\Wendy\20230404\sample 11_000001.d	Acquisition Date	4/4/2023 11:19:49 AM
Method	lmw_low_DI_20211027	Operator	Admin
Sample Name	sample 11	Instrument	solariX XR
Comment			

Acquisition Parameter

Acquisition Mode	Single MS	Acquired Scans	8	Calibration Date	Tue Apr 4 10:23:39 2023
		No. of Cell Fills	1	Data Acquisition Size	1048576
Broadband Low Mass	98.3 m/z			Data Processing Size (SI)	2097152
Broadband High Mass	2000.0 m/z			Apodization	Full-Sine
Source Accumulation	0.000 sec				
Ion Accumulation Time	0.300 sec				

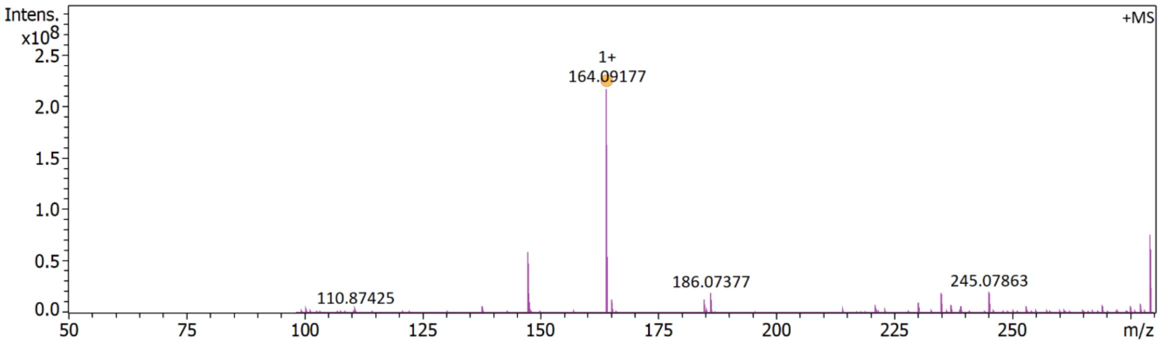

Meas. m/z	#	Ion Formula	Score	m/z	err [ppm]	Mean err [ppm]	mSigma	rdb	e⁻ Conf	N-Rule
164.091773	1	C6H14NO4	100.00	164.091734	-0.2	-0.3	3.0	1.0	even	ok
	2	C7H10N5	44.54	164.093072	7.9	7.8	12.1	6.0	even	ok

Compound 29: 3,4-Di-*O*-acetyl-5-bromo-5-deoxy-1,2-*O*-isopropylidene-α-L-tagatopyranose

^{1}H NMR (400 MHz, $CDCl_3$)

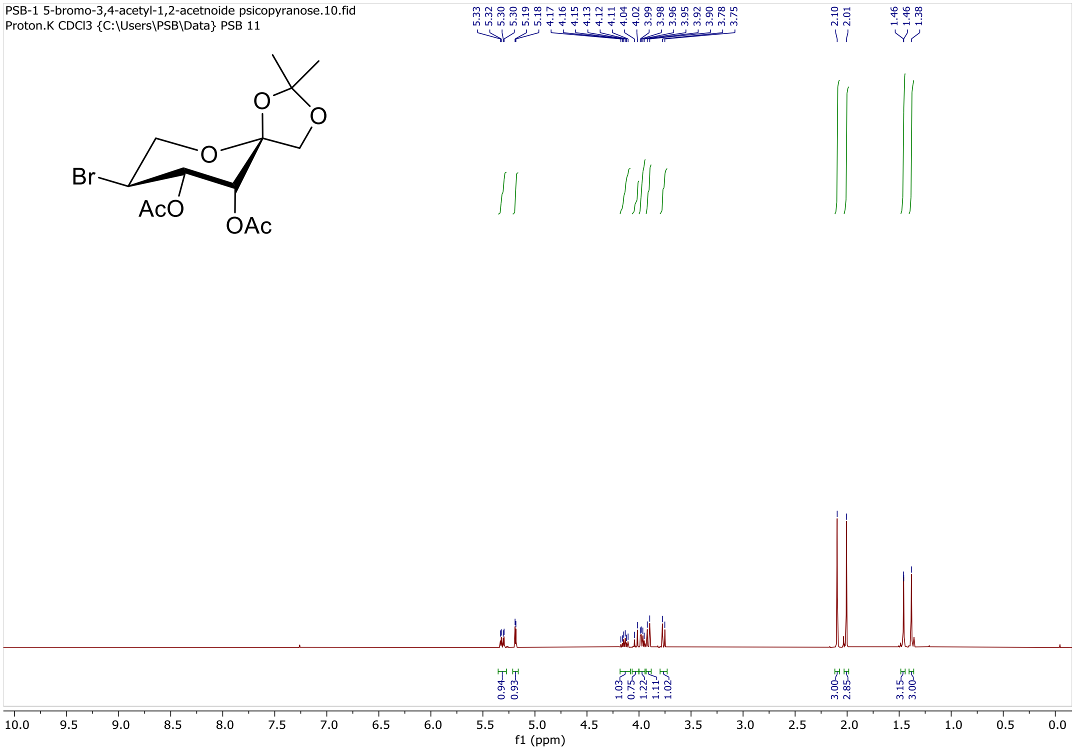

^{13}C{^{1}H} NMR (100 MHz, $CDCl_3$)

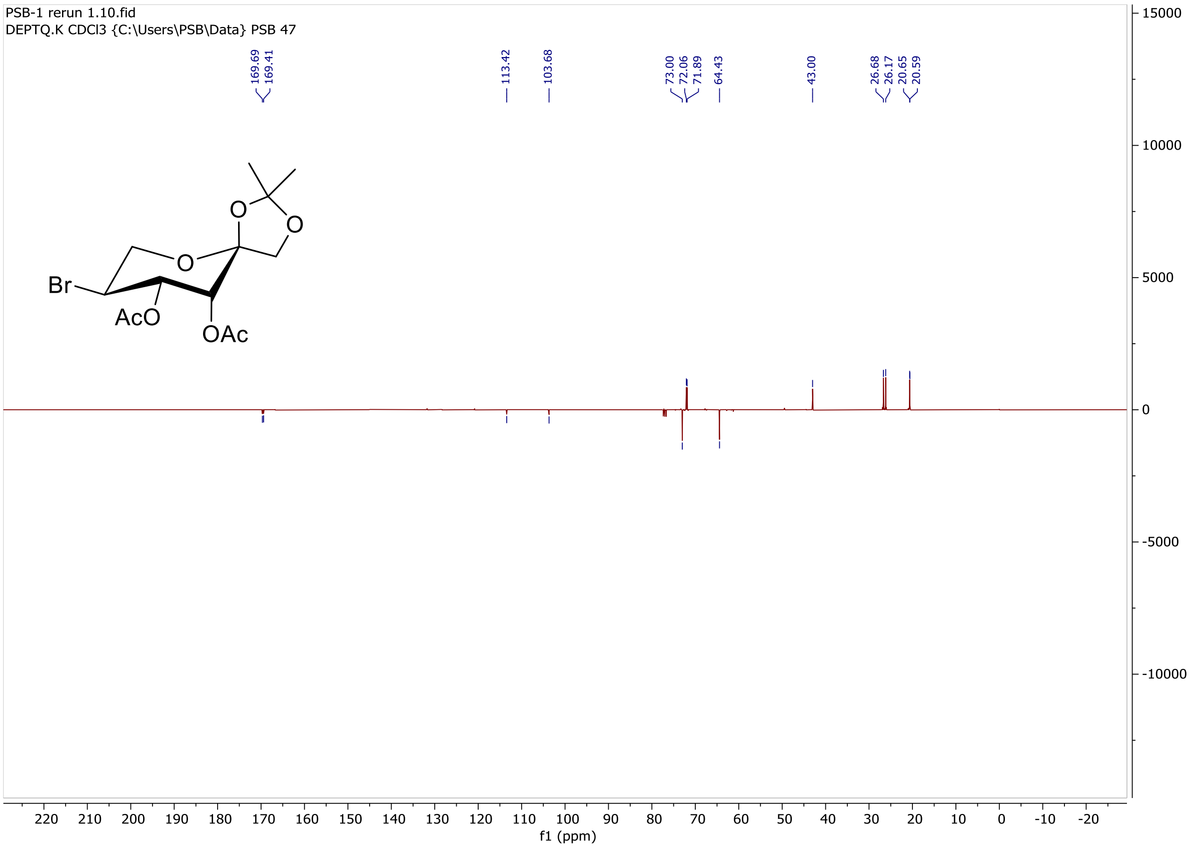

^{1}HSQC{^{13}CDEPT}

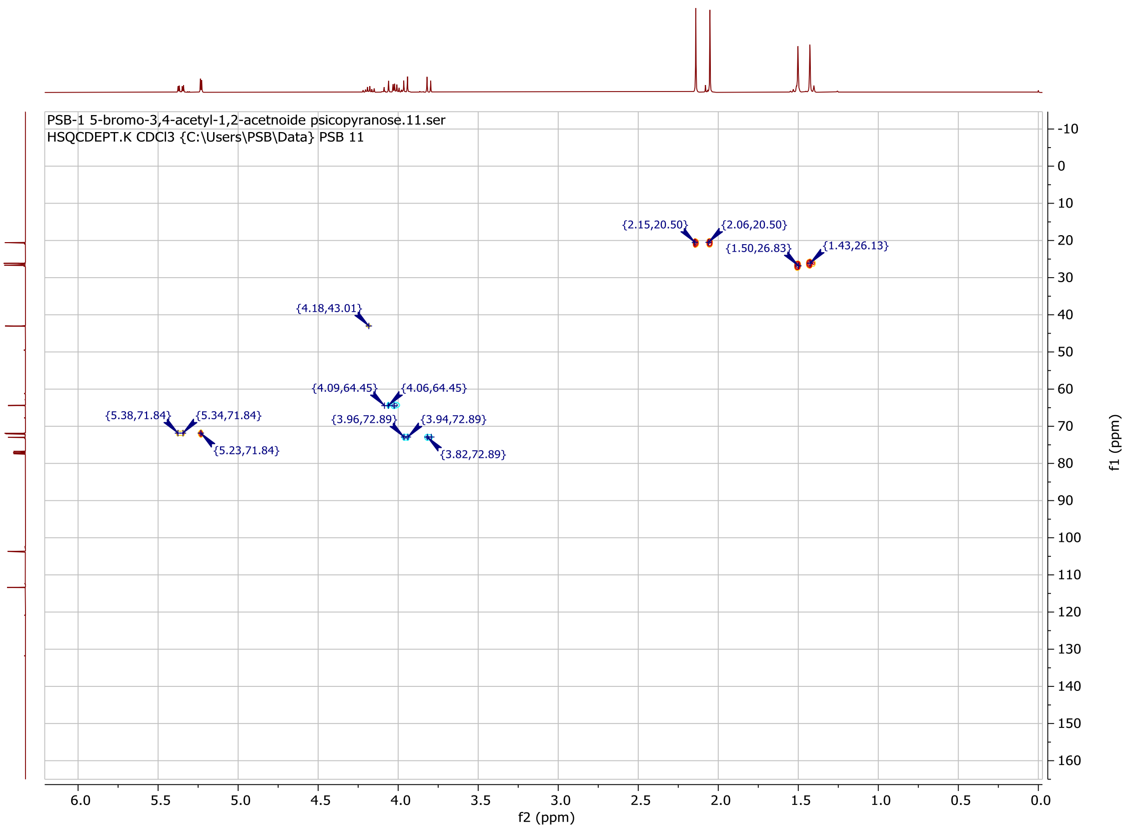

HRMS

**Name**	5-bromo tagaato inversion appel	**Data File**	2024_246.d
**Inj. Vol. (ul)**	1	**Method (Acq)**	Keele_HRMS_Positive_Long.m
**Target Formula**	C13H19BrO7	**Acq. Time (Local)**	26/04/2024 06:04:30 (UTC+01:00)
**MS Type**	QTOF	**Ionisation Type**	ESI
**Solvent**	MeOH/MeOH	**Ion Polarity**	Positive
**Result Summary**	1 qualified (1 targets)	**IRM Status**	Success

Compound Details

Cpd. 1: C13 H19 Br O7

Formula	Mass (Tgt)	Mass	RT	Score	Algorithm	Diff (Tgt, ppm)
C13 H19 Br O7	366.0314	366.0308	5.178	94.20	FBF	-1.61

Compound ID Table

Formula	Species	ID Source	Flag Severity	Flags
C13 H19 Br O7	(M+NH4)+ (M+Na)+ (2M+Na)+	FBF	Warning	No H adduct

Compound Chromatograms (overlaid)

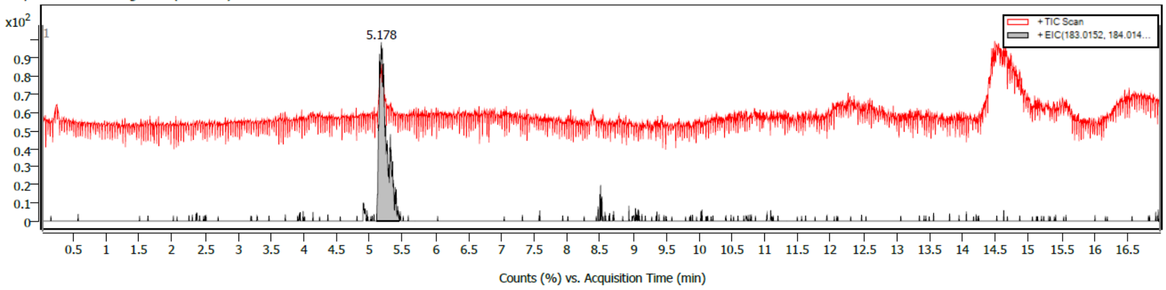

Compound Spectra (overlaid)

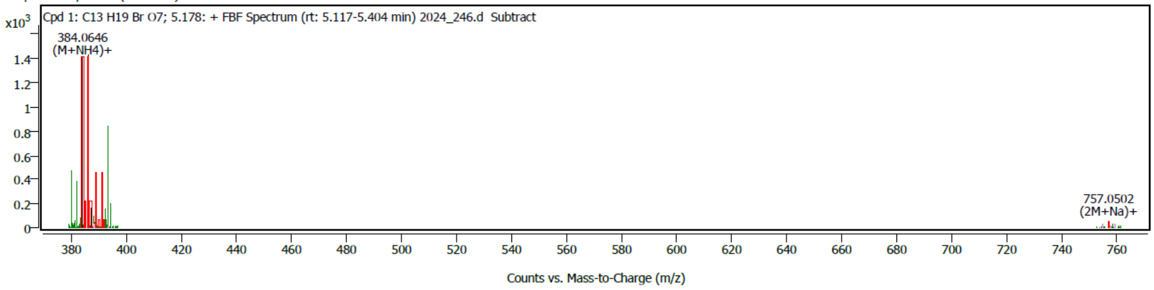

Spectrum Peaks

Ion Species	m/z	m/z (Calc)	Z	Abund	Diff (ppm)	Height %	Height % (Calc)	Formula	Sat.
(M+NH4)+	384.0646	384.07	1	1412	-1.79	98.70	100.00	C13H19BrO7	
(M+NH4)+	385.0680	385.07	1	155	-1.23	10.81	14.96	C13H19BrO7	
(M+NH4)+	386.0633	386.06	1	1430	-0.24	100.00	99.76	C13H19BrO7	
(M+NH4)+	387.0640	387.07	1	160	-6.72	11.20	14.81	C13H19BrO7	
(M+Na)+	389.0191	389.02	1	452	-3.84	100.00	100.00	C13H19BrO7	
(M+Na)+	391.0186	391.02	1	435	-0.34	96.32	99.70	C13H19BrO7	
(M+Na)+	392.0194	392.02	1	36	-7.02	7.93	14.40	C13H19BrO7	
(2M+Na)+	757.0502	757.05	1	53	-0.10	100.00	100.00	C13H19BrO7	

Compound 30: 3,4-Di-*O*-acetyl-5-bromo-5-deoxy-1,2-*O*-isopropylidene-α-L-psicopyranose

^{1}H NMR (400 MHz, $CDCl_3$)

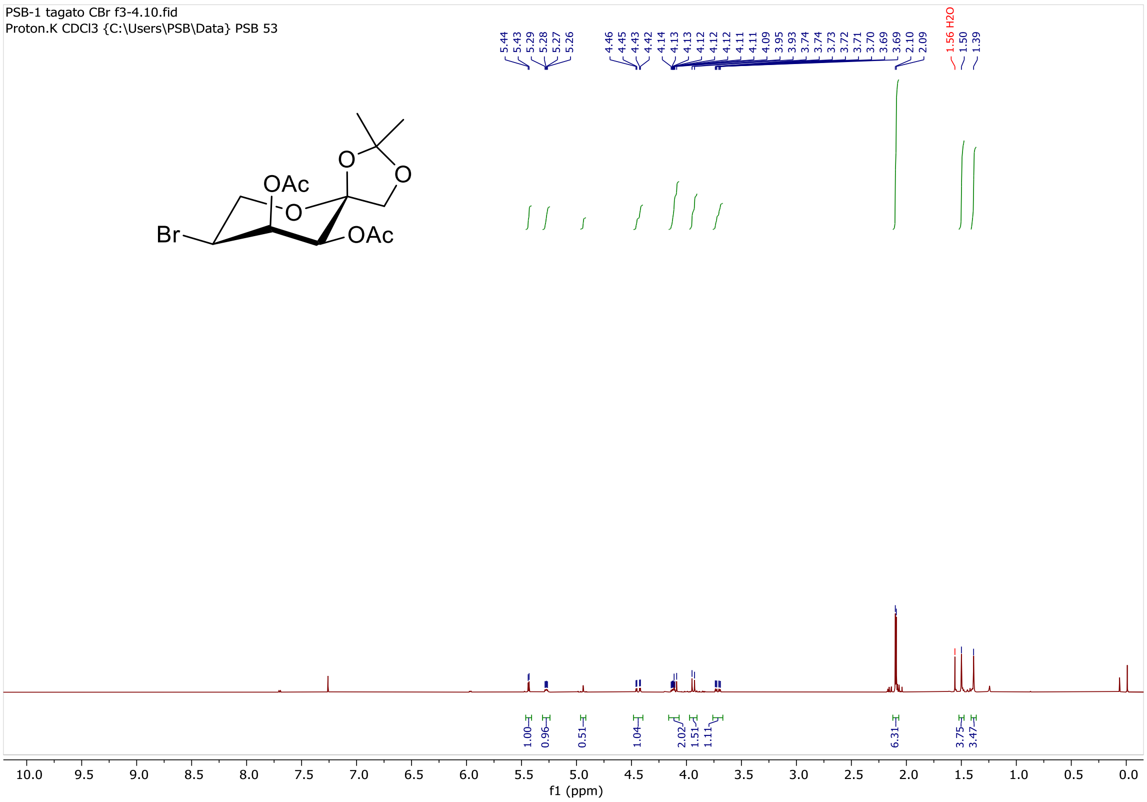

^{13}C NMR (100 MHz, $CDCl_3$)

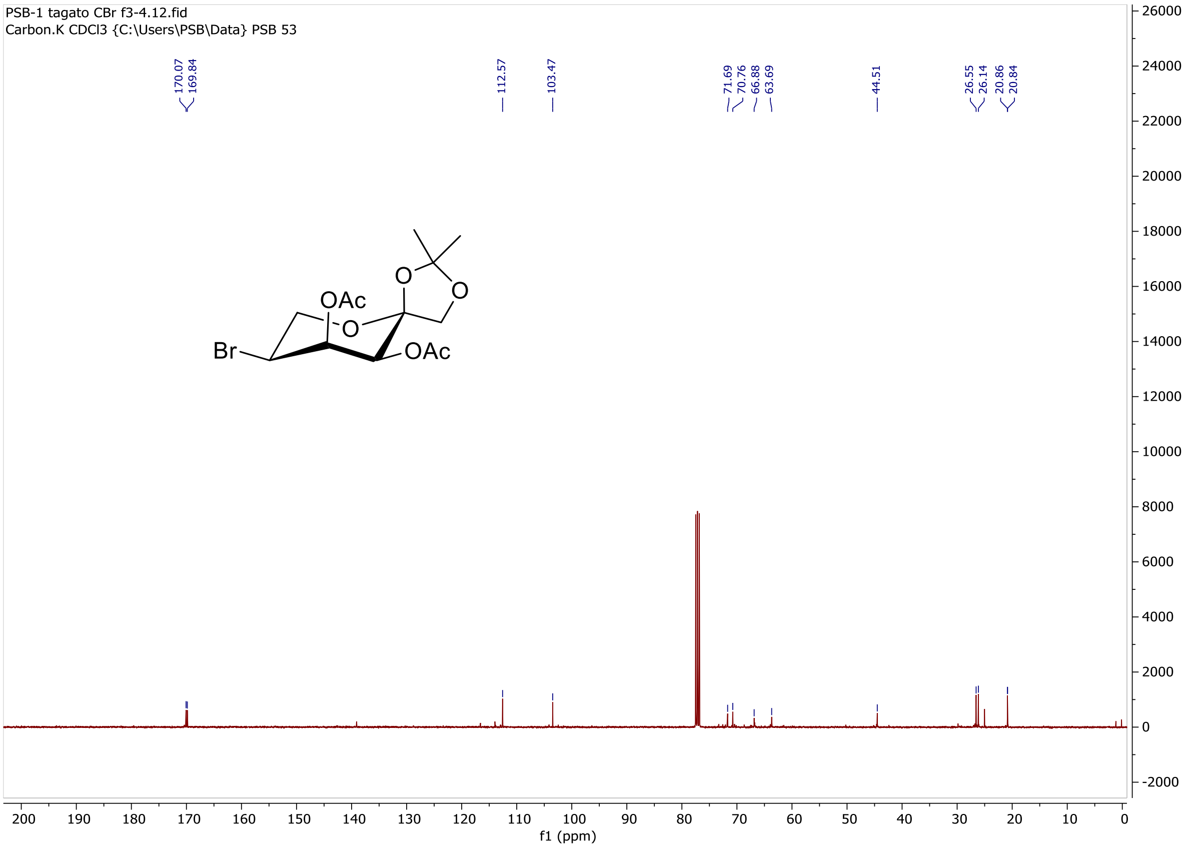

$^{1}HSQC\{^{13}CDEPT\}$

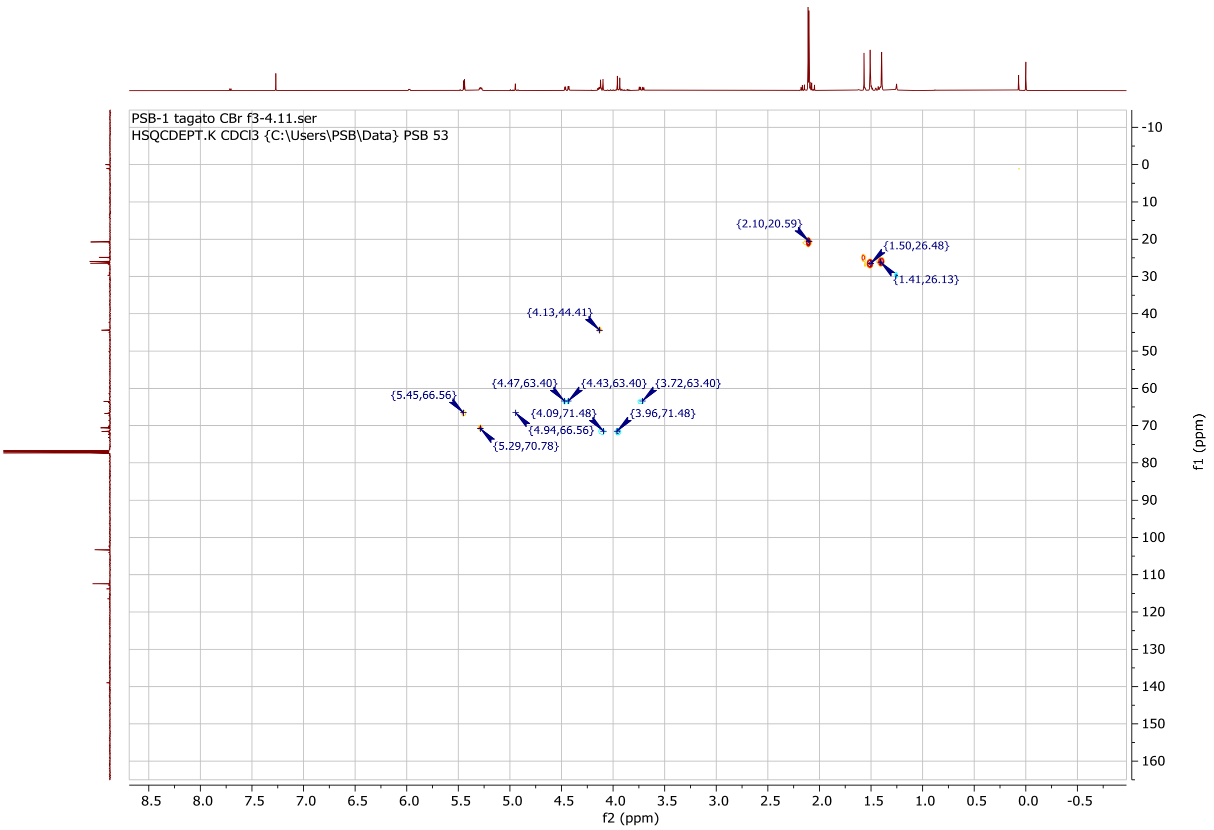

HRMS

**Name**	5-bromo-3,4 acetyl 1,2 acetnoide l-psicopyranose	**Data File**	2024_081.d
**Inj. Vol. (ul)**	1	**Method (Acq)**	Keele_HRMS_Positive.m
**Target Formula**	C13H19BrO7	**Acq. Time (Local)**	05/03/2024 23:05:04 (UTC+00:00)
**MS Type**	QTOF	**Ionisation Type**	ESI
**Solvent**	DCM/MeCN	**Ion Polarity**	Positive
**Result Summary**	1 qualified (1 targets)	**IRM Status**	Success

Compound Details

Cpd. 1: C13 H19 Br O7

Formula	Mass (Tgt)	Mass	RT	Score	Algorithm	Diff (Tgt, ppm)
C13 H19 Br O7	366.0314	366.0301	2.244	75.48	FBF	-3.64

Compound ID Table

Formula	Species	ID Source	Flag Severity	Flags
C13 H19 Br O7	(M+NH4)+ (M+Na)+ (2M+NH4)+ (2M+Na)+	FBF	Warning	No H adduct

Compound Chromatograms (overlaid)

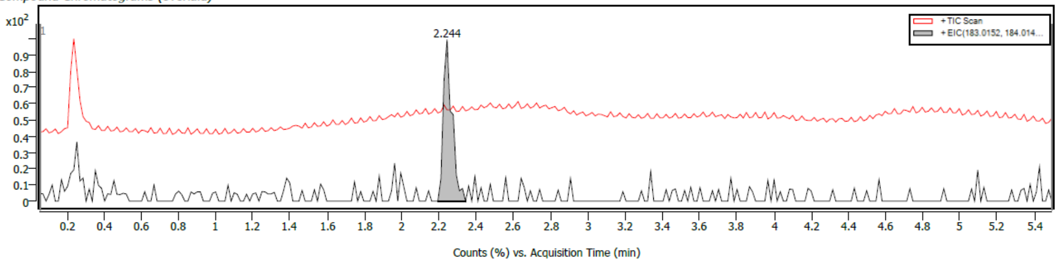

Compound Spectra (overlaid)

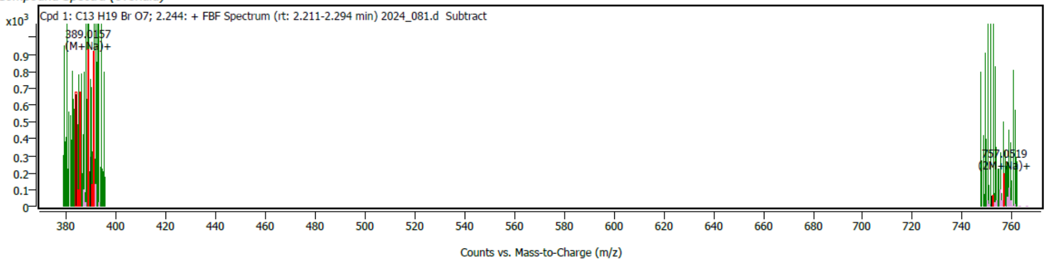

Spectrum Peaks

Ion Species	m/z	m/z (Calc)	z	Abund	Diff (ppm)	Height %	Height % (Calc)	Formula	Sat.
(M+NH4)+	384.0643	384.07	1	660	-2.42	97.53	100.00	C13H19BrO7	
(M+NH4)+	385.0656	385.07	1	71	-7.65	10.45	14.96	C13H19BrO7	
(M+NH4)+	386.0636	386.06	1	677	0.46	100.00	99.76	C13H19BrO7	
(M+Na)+	389.0157	389.02	1	911	-12.60	100.00	100.00	C13H19BrO7	
(M+Na)+	390.0244	390.02	1	290	0.95	31.82	14.55	C13H19BrO7	
(M+Na)+	391.0198	391.02	1	775	2.55	85.02	99.70	C13H19BrO7	
(2M+NH4)+	752.0805	752.09	1	62	-19.11	100.00	100.00	C13H19BrO7	
(2M+Na)+	757.0519	757.05	1	195	2.18	100.00	100.00	C13H19BrO7	

MassHunter Qual 10.0
(End of Report)
